# Heterogenous long-term health and social outcomes of type 1 diabetes: a full population 30-year observational cohort study

**DOI:** 10.1093/aje/kwaf028

**Published:** 2025-02-11

**Authors:** Aapo Hiilamo, Niina Metsä-Simola, Philipp Dierker, Pekka Martikainen, Mikko Myrskyla

**Affiliations:** Laboratory of Population Health, Max Planck Institute for Demographic Research, Rostock, Germany; Max Planck—University of Helsinki Center for Social Inequalities in Population Health, Rostock, Germany; Max Planck—University of Helsinki Center for Social Inequalities in Population Health, Helsinki, Finland; Max Planck—University of Helsinki Center for Social Inequalities in Population Health, Rostock, Germany; Max Planck—University of Helsinki Center for Social Inequalities in Population Health, Helsinki, Finland; Helsinki Institute for Demography and Population Health, University of Helsinki, Helsinki, Finland; Laboratory of Population Health, Max Planck Institute for Demographic Research, Rostock, Germany; Max Planck—University of Helsinki Center for Social Inequalities in Population Health, Rostock, Germany; Max Planck—University of Helsinki Center for Social Inequalities in Population Health, Helsinki, Finland; Helsinki Institute for Demography and Population Health, University of Helsinki, Helsinki, Finland; Max Planck—University of Helsinki Center for Social Inequalities in Population Health, Rostock, Germany; Max Planck—University of Helsinki Center for Social Inequalities in Population Health, Helsinki, Finland; Helsinki Institute for Demography and Population Health, University of Helsinki, Helsinki, Finland; Laboratory of Population Health, Max Planck Institute for Demographic Research, Rostock, Germany; Max Planck—University of Helsinki Center for Social Inequalities in Population Health, Rostock, Germany; Max Planck—University of Helsinki Center for Social Inequalities in Population Health, Helsinki, Finland; Helsinki Institute for Demography and Population Health, University of Helsinki, Helsinki, Finland

**Keywords:** type 1 diabetes, causal forest, register research

## Abstract

Type 1 diabetes (T1D) is known to have adverse long-term health and social outcomes, but the modifying factors are largely unknown. We investigate to what extent T1D outcomes are modified by area-, household-, and individual-level social and economic characteristics in Finland. National registers from 1987 to 2020 were used to identify all 3048 children with T1D diagnosed at ages 7-17 years and matched controls (*n* = 78 883). Using causal forests, we estimated the average association between T1D and adult health, social, and economic outcomes at ages 28-30 years, and the modifying roles of more than 30 covariates. Individuals with T1D were more likely to be deceased (2.3% vs 0.9% in the control group), to use antidepressants (17% vs 13%), and to be unpartnered (36% vs 32%), and had more months of unemployment (1.18 vs 1.02) and lower annual income (25 697 euros vs 27 453 euros), but not significantly lower educational attainment (10.8% vs 10.3% with only basic education). Type 1 diabetes had a heterogenous association with all outcomes except mortality and income, but no specific population subgroup was vulnerable across all outcomes. However, women with T1D had particularly high rates of antidepressant use, and individuals from low socioeconomic families were more likely to be unpartnered.

## Introduction

The global prevalence of type 1 diabetes (T1D) has increased substantially,^1^ affecting 1.5 million children and adolescents in 2021, and is forecasted to double by 2040.^2^ Despite advances in diabetes management, the life expectancies of people with T1D are significantly lower than those of the general population, with substantial between-country variation. In Finland, the life expectancy at age 20 years of people with T1D is some 10 years shorter than that of the general population^3^; a difference that is much smaller than the high-income countries’ average.^2^ In addition, the risk of depression remains substantially higher for people with T1D than for the general Finnish population.^4^ International evidence shows that the long-term adulthood social outcomes of individuals with T1D include lower adulthood income,^5^^,^^6^ lower rates of employment,^7^^,^^8^ and higher rates of childlessness.^9^ The evidence for the association of T1D with educational outcomes is mixed: lower school grades and graduation rates have been reported in Denmark^10^ and Sweden,^11^ but Australian^12^ and Welsh^13^ register studies found no differences by T1D status (see the literature review in Text S3). This divergence in mortality and education outcomes across countries suggests that contextual factors may play a key role in determining the impact of T1D on people’s life courses.

However, little is known about within-country variations in the consequences of T1D in adulthood by cohorts and area-, household-, and individual-level factors. Managing T1D requires lifelong daily monitoring of blood glucose and lifestyle adaptations, but families are likely to have different abilities to cope with these requirements. For example, child poverty is associated with T1D complications,^14^ and lower income is associated with worse T1D management adherence.^15^ Moreover, the burden of self-management, such as monitoring blood glucose, and the fear of complications can cause mental stress and make schooling difficult.^16^ Type 1 diabetes-related stigma might also have complex intersecting effects on long-term outcomes.^17^^,^^18^ However, recent cohorts may cope better, have increased access to knowledge, and experience less stigmatization because of improvements in medical care. These potential variations in T1D outcomes have not been studied using reliable population-level data. Yet identifying those individuals whose life courses are most impacted by T1D is important to design targeted care and support.

In this study, we investigate the extent to which the long-term impact of T1D differs by modifying factors in Finland. The country has the highest prevalence rate of T1D globally,^19^ for reasons that has been previously speculated to do with “an interaction between changes in lifestyle, the living environment, and predisposing genes.”^20^ We focus on people diagnosed with T1D during their school years and population matched controls to investigate their adult outcomes up to 23 years later. We apply an outcome-wide approach,^21^ with 6 adult outcomes reflecting complementary domains of life as conceptualized in the WHO quality of life instrument^22^: physical (mortality), psychological (antidepressant purchase), level of independence (employment), social relationships (partnership), and environment (income and education). Following the social ecological model of disease development, according to which the consequences of diseases are formed through an interplay of numerous layers,^23^ we examine the modifying role of 38 economic, social, and health variables at the area (postcode area and region), household, and individual levels.

## Methods

We used Finnish total population register data, which allowed us to reliably identify children with and without diagnosed T1D and link them to their parents, households, and postcode areas; and then to ascertain a rich set of covariates, potential modifiers, and outcomes. In the Finnish register data, information from different administrative sources were combined via (pseudonymized) personal, family, and household identification numbers. We combined annual socioeconomic and demographic data from Statistics Finland (1987-2020), information on specialized healthcare from the Finnish Institute for Health and Welfare (1970-2015), and information on reimbursements and special refund rights for prescription medication purchases from the Social Insurance Institution (special refund rights 1970-2019 and prescription register 1994 onwards-). These data were saved and analyzed in a remote secure access server, and approved by the Ethics Committee of Statistics Finland (TK/3343/07.03.00/2023) and Findata (THL/5674/14.06.00/2023).

We identified people who were diagnosed with T1D diabetes during school ages^7^^-^^17^ from a source population of all people born between 1981 and 1990 and residing in Finland in the calendar year they turned six (*n* = 642 501). We first excluded persons who were diagnosed with T1D (see identification below) before the calendar year they turned 7 years (1635 excluded), had no data on parental or household covariates (5202 excluded), died before the year they turned 18 years (1324 excluded), or immigrated before the year they turned 18 years (7409 excluded).

From the remaining sample, we identified 3048 persons with a new TD1 diagnosis before the calendar year they turned 18 from 3 national registers: the special reimbursement register (*n* = 2747), hospital visits records (277 identified), and insulin prescription records (24 identified; Figure S1). We used antidiabetic medications reimbursement codes in the special reimbursement register.^24^ For specialized care data, we used expressions for *ICD-9* codes of 250#B and 250##B and *ICD-10* codes of E10 in primary or secondary diagnoses. For medicine prescription data from 1995 onward, we used insulin ATC codes A10A*. The year of diagnosis was defined as the first record in any of these registers (the distribution of the diagnosis age is provided in Figure S2). These three databases are reliable in identifying people with T1D.^24^

To reduce computational demands, we matched each person with T1D with up to 26 controls without T1D based on their region of residence, sex, and birth year (fewer controls were selected if the stratum size was smaller). We used Stata’s pseudorandom number generation to select the controls randomly within each stratum. The analysis consisted of 78 883 persons in the comparison group.

### Outcomes

We measured mortality from the death records of Statistics Finland until the calendar year of the person’s 30th birthday. All other outcomes were measured during a 3-year period from the calendar year of the person’s 28th birthday to the calendar year of the person’s 30th birthday to increase the robustness of the measurements and address random fluctuations in them.

Data on purchases of antidepressants were obtained from the prescription register of the Social Insurance Institution and included the ATC codes N06A (antidepressants) and N06CA (antidepressants in combination with psycholeptics). We identified individuals with at least one antidepressant purchase to measure potential effects over a longer period of time (this outcome was not available for the 1990 cohort because the prescription register was available until 2019 only). Information on partnership, education, employment, and income was obtained from Statistics Finland Living in a partnership was defined as living with a cohabiting or married partner at the end of at least 1 year during the 3-year outcome measurement period, to capture continuous singlehood. Individuals without a secondary or higher educational qualification were categorized as having basic education. Unemployment was measured as the number of months each individual was unemployed during the 3-year measurement period, divided by three. Finally, income included all sources of taxable income each individual had during the 3-year period (this outcome was not available for the 1990 cohort), divided by three. Annual income was rounded to hundreds and top coded by Statistics Finland for privacy concerns. These outcomes, except mortality, were conditional on being alive and resident in Finland during the 3-year measurement period.

### Modifiers

We explored the role of 38 covariates as potential modifiers of the association of T1D with adult outcomes. All covariates were considered as sources of both confounding and modification, and were measured during the calendar year the person turned 6 years, and thus before the T1D diagnosis.

Individual-level covariates included sex, first language (Finnish vs other), and number of specialized healthcare visits at ages 2-6 years. Parent-level covariates (measured separately for mothers and fathers) included age, highest educational level (5 categories), educational field (health vs other), employment (yes or no), number of specialized healthcare visits at ages 2-6 years of the child, and marital status (married vs other, for mothers only). For individuals with missing parental information, the reference group was imputed and an additional variable of missing parent was included in the modeling.

Household-level covariates included having another person with diabetes in the household as well as measures of family structure and socioeconomic resources. Covariates for family structure included number of co-resident children, household size (number of persons), and family type (single parent vs other). Measures for socioeconomic resources included taxable assets, debts, any income from social assistance, any income from the child home-care allowance, any income from unemployment benefits, salary income, business income, any pension income, social insurance benefits (health and parental allowances), living in an overcrowded household, housing type (flat vs other), and homeownership (owner vs other).

Postcode-level variables included the share of foreign-born, the share of unemployed, the share of adults with less than a secondary education, and the share of Swedish speakers (which is often considered a proxy for area-level social capital). We also included the region (17 variables) and the remoteness of the area (7 categories from inner urban area to sparsely populated rural area). We included the birth year to capture macro-level changes. See Text S2 for the variables and their data sources.

### Methods

Our estimand of interest is the conditional average treatment effect (CATE) of T1D on selected adult outcomes. The individual effect—never observed—is the difference between two potential adult outcomes for the same person: outcome if the person has T1D and outcome if they do not. Conditional average treatment effect is the average of these effects conditional on one’s modifier values. Inspecting the distribution of CATEs across different modifier values allows us to understand whether there is meaningful variation in the long-term outcomes of T1D. We take a data-driven, explorative causal forest (CF) approach to estimating CATE. CF is a machine learning technique based on random forests,^25^ applied to causal settings where the prediction target is CATE.^26^ Causal forest consists of causal trees that break the data into leaves to maximize differences in estimated CATEs across the leaves while considering the sample sizes on both sides of the split. Large numbers of trees are grown (for different random subsamples), from which adaptive forest weights for individual observations are calculated as the number of times other observations fall in the same leaf. The final CF-estimated CATE for each observation given this person’s modifier values is calculated from the forest-weighted residual-on-residual regressions. All associations are modeled on an additive scale. We provide a more detailed description in Text S1.

Given our large number of covariates, we train 2 forests.^27^ The first is a pilot forest, which selects the most relevant modifiers and excludes the weak ones. We use covariate importance, which is the weighted number of times a variable is used to make a split in the forest, as a measure of modifier relevance. The second, final forest includes the modifiers that are above the mean importance in the pilot forest. We train both forests with all tuning parameters. From the CF estimates, we also calculate the average effect on the treated, ie, the differences in outcomes if all individuals with T1D do not have T1D compared to the real situation. We use augmented inverse probability weighting, which is robust to misspecifications on either outcome or treatment models.

We assess the evidence for effect heterogeneity, ie, significantly different CATEs for the potential modifiers, by dividing the sample into ranked quartiles according to the estimated CATEs for each outcome and then inspecting the characteristics of these groups. To avoid overfitting, we assign these ranks based on forests to which the assigned observations do not contribute. This is done by dividing the sample into 10 random subsets and iteratively running the models on 9 subsets while ranking the 10th, unseen subset based on the 9 subsets forest.^28^ This process is repeated to assign ranks to all 10 subsets. We then explore the sources of potential variation in CATEs by calculating means of the covariates at each rank. We call those assigned to the first ranked CATE quartile the “low effect group” (least susceptible to adverse effects of T1D) and those assigned to the last ranked CATE quartile the “high effect group” (most susceptible to the adverse effects of T1D). We calculate the standardized unadjusted differences between these two groups to summarize the covariate composition of the people with estimated high and low effects. We also report variable importance as a measure of heterogeneity (Figure S3) and plot covariates against the predicted CATEs (Figures S4-S9). Stata/Mp 18.0 was used for the data management and R version 4.3.1 and grf package (version 2.3.2) for the CF analysis.

## Results

The distributions of the covariates between people diagnosed with T1D and the matched controls were similar, except for those of any type of diabetes in the household, previous hospital visits, and sibling count (Table 1). The distributions of the covariates were similar in the analyzed sample with the general population, except that the share of women were higher in the general population (Table S1).The T1D group had worse adult outcomes (Table 2): they had a higher death rate by age 30 (2.3% vs 0.9%, *P* = 2.9 ×10^−15^), a higher rate of antidepressant use (17.1% vs 13.1%, *P* = 1.7 ×10^−9^), and a higher likelihood of not having a partner (36% vs 32%, *P* = 3.2 ×10^−6^). The difference in the percentage with only a basic level of education was insignificant (10.8% vs 10.3%, *P* = .404). The differences in months spent in unemployment (1.18 vs 1.02 months, *P* = 2.1 ×10^−4^) and in income (25697e vs 27453e, *P* = 4.6 ×10^−14^) were substantial.

**Table 1:** Characteristics of the type 1 diabetes (T1D) study population, the matched sample, and the full population.

**Term**	**T1D group**	**Matched group**	**Full population**
Number of observations	3048	78 883	626 931
Any social assistance	0.10	0.12	0.11
Birth year	1985.75	1985.75	1985.46
Father working	0.83	0.83	0.84
Homeowner	0.75	0.75	0.76
Hospital visits 4-6	0.47	0.39	0.51
Household assets	20445.39	21699.77	21237.23
Household earnings	25680.74	26571.45	26300.09
Household size	4.47	4.55	4.55
Mother working	0.71	0.71	0.71
Single parent	0.11	0.12	0.11
Women	0.43	0.42	0.49

Finnish register study. Means/fractions. Selected variables. All covariates are shown in Table S1.

**Table 2:** Mean outcomes of the type 1 diabetes group and the matched sample. Means/fractions.

**Outcomes at age 28-30 years (the calendar year)**	**T1D group**	**Matched sample**	**Full population**	***P*-value for matched vs T1D difference** ^**a**^
Mortality	2.3%	0.9%	0.9%	2.9 ×10^-15^
Antidepressant use	17.1%	13.1%	13.6%	1.7 ×10^-9^
No partnership	35.8%	31.8%	30.9%	3.2 ×10^-6^
No education	10.8%	10.3%	10.0%	0.404
Unemployment months	1.18	1.02	1.01	2.1 ×10^-4^
Income	25 697	27 453	27 198	3.2 ×10^-6^

a *t*-test for continuous variables and chi2 for dichotomous.

**Figure 1:**
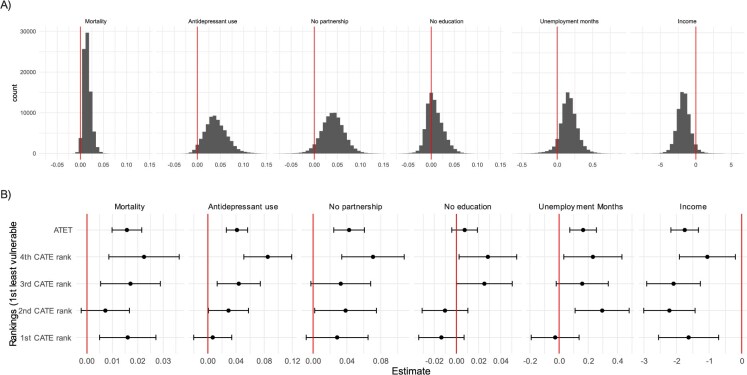
Distribution of estimated conditional average treatment effects (CATEs, panel A) from causal forest (CF) and the average treatment effects on the treated (ATET) and average treatment effects for the CATE four rank groups (panel B). Separate models for each outcome; estimated probability/mean differences; income is in thousands.

Figure 1 panel a shows the distribution of the conditional average treatment effects (CATEs), and panel b the effects in ranked CATE fourths and the average effects. According to the ranked CATE fourths, T1D showed a differential association with antidepressant use, not living with a partner, having only basic education, and months spent in unemployment. The evidence for the modified association of T1D with mortality was uncertain due to the low number of deaths, and the evidence for the association of T1D with income was unclear. In the high effect group, T1D was linked to a 8.5% point higher risk of antidepressant use (5.1-11.9). This association was insignificant in the group with the lowest CATE rank (1% point [–2 to 3.4]). For the partnership outcome, the low effect group did not show a significant association (2.8% points [–1 to 6.5]), but for the other three ranked fourths, the associations were positive, with the high effect group having a 7.1% point higher probability of not living with a partner (3.4-10.8). For the education outcome, two ranked fourths of the sample showed no association, while the high effect group showed a slightly higher risk of having only basic education (2.8% point [0.2-5.4] probability difference). For the unemployment outcome, T1D was not linked to more months spent in unemployment months in the low effect group (–0.03 months –0.19 to 0.14), while this value was 0.23 months in the high effect group (0.03-0.43).

**Figure 2:**
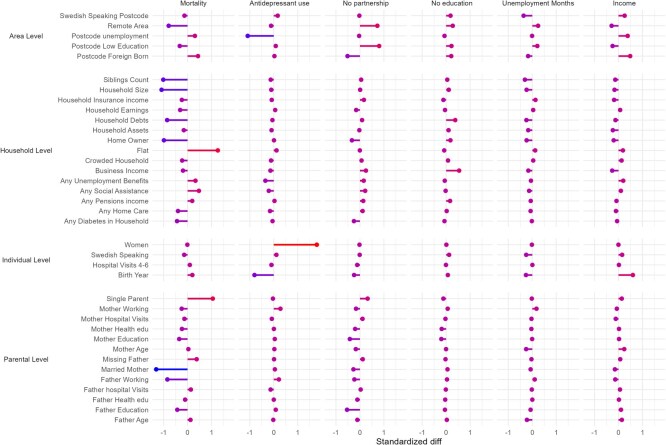
Standardized unadjusted mean differences in covariates between the first (low effect; the least vulnerable) and the fourth (high effect; the most vulnerable) CATE ranked group by outcome. A higher value indicates that the value is higher (or more prevalent) in the most vulnerable group. Finnish register study. CATE groups are estimated using CFs.

For mortality, the CATE groups differed in terms of their socioeconomic characteristics, with childhood homeownership, parental employment, and married parents being more prevalent in the low effect group (Figure 2). For antidepressant use, the CATE groups differed little in terms of household- and parental-level variables, but women and older cohorts were more likely to be in the high effect group. Some 82% of those in the high effect group were female (compared with 15% in the low effect group; Tables S2-S7). For the partnership outcome, individuals in the high effect group were more likely to be from areas with lower educational levels, to have parents with lower educational levels, and to have lower household earnings. For the educational, unemployment, and income outcomes, differences in the area-, family-, and individual-level characteristics of the high and the low effect groups were less clear. Figure S1 shows the variable importance metrics, which indicated that similar modifying factors were relevant.

## Discussion

As the prevalence of T1D continues to increase,^2^ a key public health task is to ensure that people with T1D can live long and fulfilling lives. We first showed that in a Nordic welfare state context, where the life expectancy of people with T1D is one of the highest in the world, T1D is still linked to substantially higher mortality and to increased risks of purchasing antidepressants, being unpartnered, being unemployed, and having lower income. We then used a machine learning approach to demonstrate that for many of these outcomes, the associations are heterogeneous. Finally, we aimed to identify vulnerable population subgroups, but found none across all of the outcomes we studied.

Our findings confirm earlier reports that T1D is, on average, linked to adverse adult outcomes. The average effects we found on mortality and antidepressant use are in line with those reported in previous Finnish and international studies.^3^^,^^4^ For income and unemployment, we also found average associations that are similar to those reported in earlier Nordic studies.^5^^,^^29^ However, our finding of no association between T1D and later educational attainment differs from the results of earlier Danish^10^ and Swedish^11^ studies, which reported negative associations between T1D and educational outcomes. These conflicting findings are unlikely to be due to methodological differences. Although confounding is not a significant concern when studying the impact of T1D, we nevertheless adjusted for covariates using the double robust method, an approach that is similar to those used in the previous studies. Thus, the observed divergence in results is likely to reflect true contextual effects. The Finnish educational system, in close coordination with healthcare and education professionals, may provide the support children with T1D need.

The key contribution of this study is to show that average associations can hide substantial variation in outcomes. Previous studies that explored the role of modifying factors in T1D outcomes had small sample sizes; tested only one or a small number of modifying factors; and, in case of mortality, often lacked a comparison group (Text S3). Our study is the first to investigate area-, household-, and individual-level modifying factors with an outcome-wide study design. While the low number of deaths made it difficult to assess variation in mortality, for other outcomes we observed heterogeneity in the associations. However, for education, unemployment, and income, this heterogeneity did not reveal any easily identifiable vulnerable subgroups. These findings conflict with those of a recent Danish study, which, using a similar machine learning approach, reported that the association between T1D and adult income was stronger among those with low parental educational levels.^5^ This may be related to true country differences, given that the study designs and the numbers of people with T1D included were fairly similar. In Denmark, higher parental education was assumed to facilitate the adaptation of new medical technologies, which might have improved the income prospects of their children.^5^

It is well established that antidepressant use is generally higher among people with T1D,^4^ but our machine learning approach provided us with a novel finding that this risk is more pronounced among women. This result is consistent with evidence showing higher levels of diabetes-related distress^30^ and other comorbidities^31^ among females. While our study cannot test the underlying mechanisms, a potential explanation for this is that the response to stress is gendered, in that women are more likely to internalize their stress while men are more prone to externalizing symptoms.^32^ Women are overall more likely than men to have depression^33^ and they are more likely to use psychotropic medications,^34^ even after adjusting for depression.^35^ Future studies are needed to examine whether T1D poses more strain to women than men, calling for more support measures targeted to women, or whether women are overrepresented in the high-risk group because they are more likely to use antidepressants. Interestingly, we did not find a modifying role of sex for the other outcomes. This is, however, consistent with a Swedish study that found no substantial sex differences in the economic outcomes of people with T1D.^7^

A link between T1D and childlessness was established in prior studies,^9^^,^^29^ but our study adds to these findings by showing lower partnership rates among people with T1D. Furthermore, this association seemed to be stronger among those with lower childhood family resources. While our design does not explain why this is the case, it is possible that higher socioeconomic groups are able to offset their children’s higher risk of being unpartnered through, for example, social support or direct monetary transfers.

Based on the social ecological model of disease development, we expected to find that different layers of the social environment would together define certain population subgroups that are vulnerable across all outcomes. However, somewhat surprisingly, this was not the case. Thus, our findings suggest that for people with T1D, the Finnish welfare and healthcare system provides quite equitable outcomes across subpopulations. Nevertheless, in terms of antidepressant use, improved support could be beneficial, particularly for females; and in terms of social relationships and mortality, additional support could be helpful for children growing up in families with low household resources.

Due to between-country differences in healthcare and advances in medical technology, it is important to study key modifying factors in other country contexts and in more recent time periods. We also note that our measure of partnership may be incomplete as it only captured cohabiting and married couples, and that our income measure may be inaccurate due to fluctuations in income that could result from family formation. The results allow causal interpretation from T1D to adult outcomes under the assumption of no residual confounding. Although a key strength of our study is the large number of covariates, such as parental health history, that we were able to adjust for, residual confounding may still bias our estimates. Given that Finland has an extraordinarily high prevalence of T1D, our focus on this context makes the findings of this study policy-relevant. However, we note that the generalizability to other country contexts with different barriers in access to diagnosis and care and potentially lower overall quality of T1D care is limited. We expect higher heterogeneity in other contexts with larger inequalities in access to care but more studies are needed on whether it is possible to identify specific vulnerable groups in these contexts. The advantage of our register-data approach is solid identification of T1D and reliable measurement of both outcomes and covariates. However, we excluded a small number of individuals with incomplete covariate data, and there is a possibility that we misclassify people with T1D if there are mistakes in the three registers used for the identification. Due to data availability, we restricted the mortality follow-up to age 30 years. As some of the complications and health risks due to childhood T1D are likely to appear later, it is likely that we underestimate the effect of T1D on mortality. Finally, our outcomes were measured conditionally on being in the registers, that is, being in the country and alive. Given that in addition to T1D, low socioeconomic position, poor mental health, and not having a partner are likely to increase mortality risk, our findings may underestimate the overall associations between T1D and these outcomes as well as heterogeneity in these associations.

## Conclusions

Type 1 diabetes is associated with substantially higher mortality, higher probabilities of antidepressant use, lower partnership rates, higher unemployment risks, and lower income, with substantial heterogeneity in most of the outcomes. We do not find consistent modifying factors across all outcomes or easily identifiable vulnerable subgroups, suggesting that health and social institutions provide quite equitable outcomes for people with T1D. However, more support is needed for women with T1D to enable them to overcome their higher risk of antidepressant use, and more attention should be paid to young adults from low socioeconomic position families, as T1D seems to exacerbate their risk of remaining unpartnered.

## Supplementary Material

Web_Material_kwaf028

## Data Availability

The study uses data that are collected by register authorities (Statistics Finland, the Finnish Institute for Health and Welfare, and the Social Insurance Institution) and made available to researchers for specific research purposes stated in the research plan. All data used or produced by combining original data are confidential, and the researchers cannot share them with third parties. Those interested can apply for a license to use the data for scientific research from the register authorities (Statistics Finland and Findata Health and Social Data Permit Authority).
