# Supplementary material for: Heterogenous long-term health and social outcomes of type 1 diabetes: a full population 30-year observational cohort study
Source: Am J Epidemiol. 2025 Feb 11;195(1):117–25. doi: 10.1093/aje/kwaf028 (PMC12780756; doi:10.1093/aje/kwaf028)
Supplement: Web_Material_kwaf028 [file web_material_kwaf028.docx]

**Supplementary materials for Heterogenous long-term health and social outcomes of type 1 diabetes - A full population 30-year observational cohort study**

Contents

[Description of the Causal Forest 1](#_Toc168667125)

[Description of the variables 3](#_Toc168667126)

[Literature search strategy 6](#_Toc168667127)

[Supplementary figures 12](#_Toc168667128)

[Supplementary tables 23](#_Toc168667129)

## Supplementary text 1. Description of the causal forest

Here, we introduce our parameters of interest and method of estimation. We follow the notation of the potential outcome framework (1) in which each person i has two outcomes: one with T1D exposure taking value 1, 𝑌𝑖(T1D =1), and another without T1D, 𝑌𝑖(T1D =0). The individual effect is the difference between these two outcomes: 𝜏𝑖 =𝑌𝑖(T1D =1)−𝑌𝑖(T1D =0); the average treatment effect (ATE) is the mean of these individual effects in the population of interest 𝜏=𝐸[𝑌𝑖(T1D =1)−𝑌𝑖(T1D =0)]; and the conditional average treatment effect (CATE) is the average effect in a subset of that population conditional on their covariate values 𝑋: 𝜏(X)=𝐸[𝑌𝑖(T1D =1∣𝑋=𝑥)−𝑌𝑖(T1D =0∣𝑋=𝑥)]. Here, our main parameter of interest is 𝜏(X).

Our method of estimation is causal forest (CF). CF is a machine learning technique that builds on random forests but applies it in causal settings in which the prediction target is 𝜏(X) (2). CF consists of causal trees that break the data into leaves to maximize differences in estimated 𝜏(X) across the leaves. The trees place splits to covariates X recursively by using Robinson’s residual-on-residual regression. The final CF estimated CATE for each observation is calculated from the forest weighted residual-on-residual regressions. To avoid overfitting, CF selects a random fraction of the data as the training sample to determine the covariate splits and the second fraction to estimate CATEs in each of the leaves, based on a principle known as honesty. Next, we follow the notation provided in Athey et al. (3) and provide a short summary of these key phases of CF.

Under the assumption of no confounding given measured covariates 𝑋, ATE can be estimated using the equation:

𝑌𝑖=𝜏T1D𝑖+𝑓(𝑋𝑖)+𝜖𝑖, 𝐸[𝜖𝑖∣𝑋𝑖, T1D𝑖]=0

However, the functional form of confounders 𝑓(𝑋𝑖) may be unknown. This problem is addressed in CF by using residual-on-residual regressions. First, the propensity score, that is, the probability of having T1D status observed given the covariates (4) 𝑒(𝑥)=𝐸[T1D𝑖=T1D ∣𝑋𝑖=𝑥], and the conditional mean of the outcome of interest, that is, the predicted average given the covariates 𝑚(𝑥)=𝐸[𝑌𝑖∣𝑋𝑖=𝑥], are estimated using random forests. These objects are then used in a residual-on-residual regression as follows:

𝑌𝑖−𝑚(𝑥)=𝜏(T1D𝑖−𝑒(𝑥))+𝜖𝑖

varying treatment effects, that is, CATE can be included here as

𝑌𝑖=𝜏(𝑋𝑖)𝑊𝑖+𝑓(𝑋𝑖)+𝜖𝑖, 𝐸[𝜖𝑖∣𝑋𝑖,𝑊𝑖]=0

To estimate this equation, CF aims to locate covariate spaces in which 𝜏(𝑥) is constant by first calculating random forest adaptive neighborhood weights and then using these adaptive weights in the residual-on-residual regression. The adaptive weights are calculated from the causal trees. These causal trees use residual-on-residual regression, introduced above, to place splits in the covariates by maximizing nLnR(τ^L−τ^R)2, where nr is the sample on the right and τ^L is the estimated conditional effect on the left. The covariate balancing does not have to be performed again thanks to the residual-on-residual regressions. From causal trees adaptive weights, ai(x) are calculated as the frequency with which other observations fall in the same leaf, which captures how similar these outcomes are to other observations in terms of CATE. CF can be used to calculate treatment effects using an augmented inverse probability weighted (AIPW) estimator (5). We used 1000 trees and all tunning parameters.

## **Supplementary text 2.** Description of the variables

| Variable | Name | Source | Variables used | Notes |
| --- | --- | --- | --- | --- |
| Sex |  | Folk basic module | sukup |  |
| Birth cohort |  | Folk basic module | syntyv |  |
| First language |  | Folk basic module | Kieli_k |  |
| Number of health care visits past 4 years |  | Care Register for Health Care | tupva |  |
|  |  |  |  |  |
| Mother education |  | FOLK child – parents  Folk basic module | ututku_aste |  |
| Father education |  | FOLK child – parents  Folk basic module | ututku_aste |  |
| Mother education field |  | FOLK child – parents  Folk basic module | ututku_ala |  |
| Father education field |  | FOLK child – parents  Folk basic module | ututku_ala |  |
| Mother employment |  | FOLK child – parents  Folk basic module | ptoim1 |  |
| Father employment |  | FOLK child – parents  Folk basic module | ptoim1 |  |
| Mother marital status |  | FOLK child – parents  Folk basic module | sivis | Sivis=2 |
| No data on father |  | FOLK child – parents  Folk basic module |  | Defined as having missing fathers age |
| Number of health care visit mother |  | FOLK child – parents  Care Register for Health Care |  |  |
| Number of health care visits father |  | FOLK child – parents  Care Register for Health Care |  |  |
|  |  |  |  |  |
| Another person with diabetes |  | FOLK Household-dwelling unit  Care Register for Health Care  Special reimbursement register |  |  |
| Taxable assets |  | FOLK Household-dwelling unit  FOLK income | lvar |  |
| Debts |  | FOLK Household-dwelling unit  FOLK income | velaty |  |
| Any income from social assistance |  | FOLK Household-dwelling unit  FOLK income | toimtu |  |
| Ay income from home-child allowance |  | FOLK Household-dwelling unit  FOLK income | tkotihtu |  |
| Any income from unemployment benefits |  | FOLK Household-dwelling unit  FOLK income | tyotur |  |
| Salary income |  | FOLK Household-dwelling unit  FOLK income | tyotu |  |
| Entrepenour income |  | FOLK Household-dwelling unit  FOLK income | turtuo |  |
| Any pension income |  | FOLK Household-dwelling unit  FOLK income | elatulo |  |
| Insurance benefits |  | FOLK Household-dwelling unit  FOLK income | tpar |  |
|  |  |  |  |  |
| Household size |  | FOLK Household-dwelling unit | akoko |  |
| Crowded |  | FOLK Household-dwelling unit | asva | asva=3 |
| Housing type |  | FOLK Household-dwelling unit | taty | Taty=3 |
| Homeownership |  | FOLK Household-dwelling unit | hape | Hape 1 or 2 |
| number of siblings |  | FOLK Household-dwelling unit | Lkm_k | lkm_k-1 |
|  |  |  |  |  |
| Share of foreign born |  | Costum made postcode dataset  Folk basic module | posti_alue21  svaltio_k |  |
| Share of the unemployed |  | Costum made postcode dataset  Folk basic module | posti_alue21  ptoim1 |  |
| Share of adults without any education |  | Costum made postcode dataset  Folk basic module | posti_alue21  ututk_aste |  |
| Share of Swedish speaking |  | Costum made postcode dataset  Folk basic module | posti_alue21  kieli_k |  |
| Region |  | Folk basic module | mkunta |  |
| Remoteness of the area |  | Folk basic module | maka |  |
|  |  |  |  |  |
| Outcomeincome at 28-30 |  | Folk basic module | kturaha_k |  |
| Outcomeunemployment months |  | Folk basic module | tyke |  |
| Outcomebasic education |  | Folk basic module | ututku_aste |  |
| Outcomepartnerships |  | Folk basic module | peas  pety |  |
| Outcomemortality |  | Folk basic module | kuolv |  |
| Outcome: antidepressant purchase |  | Dispensed medicines reimbursable under the National Health Insurance scheme | atc | Atc codes N06CA and N06A |
|  |  |  |  |  |
| Exposure |  | Dispensed medicines reimbursable under the National Health Insurance scheme  Care Register for Health Care  Special reimbursement register | many |  |

| **Dataset** | **Codebook link** |
| --- | --- |
| Folk basic module | <https://aineistokatalogi.fi/catalog/studies/a6946178-3c4d-432e-b4bd-7b32b80932af/datasets/6da1a107-d1b1-4f2a-ac75-596619b316bd> |
| FOLK child – parents | <https://aineistokatalogi.fi/catalog/studies/a6946178-3c4d-432e-b4bd-7b32b80932af/datasets/68cdb209-6a9f-4cd8-98d7-56e70b122e5e> |
| FOLK Household-dwelling unit | <https://aineistokatalogi.fi/catalog/studies/a6946178-3c4d-432e-b4bd-7b32b80932af/datasets/50d07305-2455-4db8-a890-586db40313c0> |
| FOLK income | <https://aineistokatalogi.fi/catalog/studies/a6946178-3c4d-432e-b4bd-7b32b80932af/datasets/56e66d05-18fd-4aa2-9c91-e64032edb605> |
| Costum made postcode dataset |  |
| Dispensed medicines reimbursable under the National Health Insurance scheme | <https://aineistokatalogi.fi/catalog/studies/ff551f4e-6842-4732-962d-74499a339c46/datasets/0644617c-0bac-44a8-a15d-1724fe10e371> |
| Care Register for Health Care: | <https://aineistokatalogi.fi/catalog/studies/7567e45d-72b7-428b-be9e-510440336edf> |
| Special reimbursement register | <https://aineistokatalogi.fi/catalog/studies/ff551f4e-6842-4732-962d-74499a339c46/datasets/ce3225ab-c7ad-464f-8933-c78ab1782a6a> |

## Literature search strategy

Search string used in the research in context box. AH screened the titles and abstracts

| Ovid MEDLINE(R) ALL <1946 to May 24, 2024> | | | |
| --- | --- | --- | --- |
| 1 |  |  | Source |
| 1 | exp Diabetes Mellitus, Type 1/ | 88632 | Langendam M, Luijf YM, Hooft L, DeVries JH, Mudde AH, Scholten RJPM. Continuous glucose monitoring systems for type 1 diabetes mellitus. Cochrane Database of Systematic Reviews 2012, Issue 1. Art. No.: CD008101. DOI: 10.1002/14651858.CD008101.pub2. Accessed 25 May 2024. |
| 2 | exp Diabetic Ketoacidosis/ | 7738 | Langendam M, Luijf YM, Hooft L, DeVries JH, Mudde AH, Scholten RJPM. Continuous glucose monitoring systems for type 1 diabetes mellitus. Cochrane Database of Systematic Reviews 2012, Issue 1. Art. No.: CD008101. DOI: 10.1002/14651858.CD008101.pub2. Accessed 25 May 2024. |
| 3 | (IDDM or T1DM or T1D).tw,ot. | 24766 | Langendam M, Luijf YM, Hooft L, DeVries JH, Mudde AH, Scholten RJPM. Continuous glucose monitoring systems for type 1 diabetes mellitus. Cochrane Database of Systematic Reviews 2012, Issue 1. Art. No.: CD008101. DOI: 10.1002/14651858.CD008101.pub2. Accessed 25 May 2024. |
| 4 | ((diabet$ or dm) adj5 ((typ? adj3 (one or '1' or I)) or typ?1 or typ?I)).tw,ot. | 74513 | Langendam M, Luijf YM, Hooft L, DeVries JH, Mudde AH, Scholten RJPM. Continuous glucose monitoring systems for type 1 diabetes mellitus. Cochrane Database of Systematic Reviews 2012, Issue 1. Art. No.: CD008101. DOI: 10.1002/14651858.CD008101.pub2. Accessed 25 May 2024. |
| 5 | ((earl$ or acidos$ or juvenil$ or child$ or keto$ or labil$ or britt$ or p?ediatric) adj6 (diabet$ or dm)).tw,ot. | 53676 | Langendam M, Luijf YM, Hooft L, DeVries JH, Mudde AH, Scholten RJPM. Continuous glucose monitoring systems for type 1 diabetes mellitus. Cochrane Database of Systematic Reviews 2012, Issue 1. Art. No.: CD008101. DOI: 10.1002/14651858.CD008101.pub2. Accessed 25 May 2024. |
| 6 | (insulin$ defic$ adj6 absolut$).tw,ot. | 209 | Langendam M, Luijf YM, Hooft L, DeVries JH, Mudde AH, Scholten RJPM. Continuous glucose monitoring systems for type 1 diabetes mellitus. Cochrane Database of Systematic Reviews 2012, Issue 1. Art. No.: CD008101. DOI: 10.1002/14651858.CD008101.pub2. Accessed 25 May 2024. |
| 7 | or/1-6 | 148234 | Langendam M, Luijf YM, Hooft L, DeVries JH, Mudde AH, Scholten RJPM. Continuous glucose monitoring systems for type 1 diabetes mellitus. Cochrane Database of Systematic Reviews 2012, Issue 1. Art. No.: CD008101. DOI: 10.1002/14651858.CD008101.pub2. Accessed 25 May 2024. |
| 8 | (babies or baby or infan* or neonat* or neo-nat* or newborn* or new-born* or perinat*).ti,ab,kf. or Infant, Extremely Low Birth Weight/ or Infant, Extremely Premature/ or Infant, Large for Gestational Age/ or Infant, Low Birth Weight/ or Infant, Newborn/ or Infant, Postmature/ or Infant, Premature/ or Infant, Small for Gestational Age/ or Infant, Very Low Birth Weight/ or Infant/ | 1679752 | https://extranet.santecom.qc.ca/wiki/!biblio3s/doku.php?id=concepts:enfant |
| 9 | (boy? or boyfrien* or boyhood* or child* or fifth-grader* or first-grader* or fourth-grader* or girl? or girlfriend* or girlhood* or juvenil* or kid? or kindergarten* or minor? or minority or paediatric* or peadiatric* or pediatric* or PICU or preschool* or pre-school* or second-grader* or seventh-grader* or sixth-grader* or stepchild* or step-child* or third-grader* or toddler? or young or youngster* or youth*).ti,ab,kf. or Child/ or Child, Preschool/ | 3800002 | https://extranet.santecom.qc.ca/wiki/!biblio3s/doku.php?id=concepts:enfant |
| 10 | (adolescen* or college* or highschool* or high-school* or ((high* or secondary) adj2 (education or school*)) or preadolescen* or pre-adolescen* or preteen* or pre-teen* or puber* or pubescen* or teen* or underage? or under-age?).ti,ab,kf. or Adolescent/ or Minors/ | 2562471 | https://extranet.santecom.qc.ca/wiki/!biblio3s/doku.php?id=concepts:enfant |
| 11 | or/8-10 | 5739220 |  |
| 12 | exp Antidepressive Agents/ | 163284 | Molyneaux E, Telesia LA, Henshaw C, Boath E, Bradley E, Howard LM. Antidepressants for preventing postnatal depression. Cochrane Database of Systematic Reviews 2018, Issue 4. Art. No.: CD004363. DOI: 10.1002/14651858.CD004363.pub3. Accessed 25 May 2024. |
| 13 | exp Neurotransmitter Uptake Inhibitors/ | 158806 | Molyneaux E, Telesia LA, Henshaw C, Boath E, Bradley E, Howard LM. Antidepressants for preventing postnatal depression. Cochrane Database of Systematic Reviews 2018, Issue 4. Art. No.: CD004363. DOI: 10.1002/14651858.CD004363.pub3. Accessed 25 May 2024. |
| 14 | exp Monoamine Oxidase Inhibitors/ | 22901 | Molyneaux E, Telesia LA, Henshaw C, Boath E, Bradley E, Howard LM. Antidepressants for preventing postnatal depression. Cochrane Database of Systematic Reviews 2018, Issue 4. Art. No.: CD004363. DOI: 10.1002/14651858.CD004363.pub3. Accessed 25 May 2024. |
| 15 | (antidepress* or anti depress* or MAOI* or RIMA* or monoamine oxidase inhibit* or ((serotonin or norepinephrine or noradrenaline or nor epinephrine or nor adrenaline or neurotransmitt* or dopamine*) and (uptake or reuptake or "re‐uptake")) or noradrenerg* or antiadrenergic or anti adrenergic or SSRI* or SNRI* or NARI* or SARI* NDRI* or TCA* or tricyclic* or tetracyclic* or heterocyclic* or psychotropic*).tw,ot. | 213017 | Molyneaux E, Telesia LA, Henshaw C, Boath E, Bradley E, Howard LM. Antidepressants for preventing postnatal depression. Cochrane Database of Systematic Reviews 2018, Issue 4. Art. No.: CD004363. DOI: 10.1002/14651858.CD004363.pub3. Accessed 25 May 2024. |
| 16 | exp Depression/ | 158005 |  |
| 17 | or/12-16 | 541932 |  |
| 18 | exp Education/ | 918421 |  |
| 19 | exp Socioeconomic Factors/ | 522624 |  |
| 20 | exp Poverty/ or exp Income/ or earnings.tw. or salary.tw. | 125297 |  |
| 21 | ((romantic or couple or intimate or partner or love or marital or dating or cohabiting or same-sex or heterosexual or polyamorous) adj1 (relationship or status or partnership)).tw,ot. | 36136 |  |
| 22 | exp Unemployment/ or exp Employment/ | 102460 |  |
| 23 | or/18-22 | 1402649 |  |
| 24 | 17 or 23 | 1919117 |  |
| 25 | exp cohort studies/ or Epidemiologic studies/ or exp case control studies/ | 2869038 | Scottish Intercollegiate Guidelines Network |
| 26 | (cohort adj (study or studies)).tw. | 352193 | Scottish Intercollegiate Guidelines Network |
| 27 | Cohort analy$.tw. | 13053 | Scottish Intercollegiate Guidelines Network |
| 28 | (Follow up adj (study or studies)).tw. | 58226 | Scottish Intercollegiate Guidelines Network |
| 29 | (observational adj (study or studies)).tw. | 178576 | Scottish Intercollegiate Guidelines Network |
| 30 | Longitudinal.tw. | 346061 | Scottish Intercollegiate Guidelines Network |
| 31 | Retrospective.tw. | 813518 | Scottish Intercollegiate Guidelines Network |
| 32 | or/25-31 | 3436579 |  |
| 33 | 7 and 11 and 24 and 32 | 967 |  |

**Supplementary text 3. Literature review**

We screened 967 abstract and titles of previous studies on the long-term health and social outcomes of childhood-onset type 1 diabetes in Medline from its inception to May 28. We adapted search strings from previous Cochrane reviews and other rigorously developed filters (the search string is provided above). While the identified studies showed that people with childhood T1D had worse adult outcomes in terms of education, employment, and income, these studies relied on small sample sizes, and only a few focused on social and health outcomes simultaneously, or on how these effects were modified. We were unable to identify studies focusing on multiple interconnecting modifying factors.

**Summary of the supplementary literature review**: We aimed to identify studies on the social and health outcomes of T1D. We searched for studies comparing groups with and without T1D in terms of our outcomes of interest in the OECD countries. The identified studies showed that groups with T1D had consistently worse health and economic outcomes. The effects of T1D on educational attainment were unclear in the literature. The identified studies are narratively summarized below.

**Mortality differences**: T1D was linked to higher mortality (6). In the population with T1D, mortality rates were found to be higher for those with low than with high socioeconomic status (7,8). The socioeconomic mortality gradient was shown to be steeper for people with than without T1D in Scotland (9). However, not all studies reported SES differences in T1D mortality (10). Most previous studies on mortality differences in people with T1D did not include a control group of people without T1D.

**Depression**: People with childhood-onset T1D were found to have a higher risk of depression than the comparison group (11–14), often many years after the diagnosis (11). T1D was shown to be associated with other stress- and anxiety-related disorders as well (12). People with co-occurrence of depression and T1D had worse treatment outcomes, for example, higher hospital admission rates (15,16). Parental relationship quality was linked to decreased depressive symptoms and alcohol use among people with T1D (17). A Swedish study reported that socioeconomic factors explained the link between T1D and antidepressant use (18).

**Personal relationships**: We identified a few studies on partnerships. A Finnish study found that people with T1D had lower fertility rates (19), and a US study found that people with T1D had fewer friends (20).

**Education**: A large number of studies focused on educational outcomes (21), most of which used register-based data from the Nordic countries. Some Nordic register studies showed that people with T1D had lower academic achievement (22–24), while others found no differences between people with and without T1D (25). Studies from different countries provided conflicting results. A study from New Zealand showed that people with T1D had marginally lower achievement levels (26), and a study from Scotland found that people with T1D had a higher risk of school exclusion (27). Australian studies, by contrast, found no differences in literacy and numeracy grades between children with and without T1D (28–30).

**Employment and income**: A Swedish study found that the T1D group had lower annual earnings (i.e., 1,411 euros lower) than the group without T1D (31). A Japanese study found that people with T1D had lower income levels than their siblings (32).

**Identified previous studies on the long-term health and social outcomes of childhood-onset type 1 diabetes**

Aalto, A.-M., Uutela, A., & Kangas, T. (1996). Health behaviour, social integration, perceived health and dysfunction. A comparison between patients with type I and II diabetes and controls. Scandinavian Journal of Social Medicine, 24(4), 272–281. https://doi.org/10.1177/140349489602400408

Almeida, M. C., Claudino, D. A., Grigolon, R. B., Fleitlich-Bilyk, B., & Claudino, A. M. (2018). Psychiatric disorders in adolescents with type 1 diabetes: A case-control study. Brazilian Journal of Psychiatry, 40, 284–289. https://doi.org/10.1590/1516-4446-2017-2259

Begum, M., Chittleborough, C., Pilkington, R., Mittinty, M., Lynch, J., Penno, M., & Smithers, L. (2020). Educational outcomes among children with type 1 diabetes: Whole-of-population linked-data study. Pediatric Diabetes, 21(7), 1353–1361. https://doi.org/10.1111/pedi.13107

Berhan, Y. T., Eliasson, M., Möllsten, A., Waernbaum, I., Dahlquist, G., & on behalf of the Swedish Childhood Diabetes Study Group 2013. (2015). Impact of Parental Socioeconomic Status on Excess Mortality in a Population-Based Cohort of Subjects With Childhood-Onset Type 1 Diabetes. Diabetes Care, 38(5), 827–832. https://doi.org/10.2337/dc14-1522

Bowden, N., Dixon, R., Anderson, V., de Bock, M., Boucsein, A., Kewene-Edwards, M., Gibb, S., Kokaua, J., Palmer, O., Paul, R., Taylor, B., Vu, H., & Wheeler, B. J. (2024). Associations between type 1 diabetes and educational outcomes: An Aotearoa/New Zealand nationwide birth cohort study using the Integrated Data Infrastructure. Diabetologia, 67(1), 62–73. https://doi.org/10.1007/s00125-023-06026-y

Campbell, R. a. S., Colhoun, H. M., Kennon, B., McCrimmon, R. J., Sattar, N., McKnight, J., Wild, S. H., & Group, S. D. R. N. E. (2020). Socio-economic status and mortality in people with type 1 diabetes in Scotland 2006–2015: A retrospective cohort study. Diabetic Medicine, 37(12), 2081–2088. https://doi.org/10.1111/dme.14239

Cooper, M. N., McNamara, K. A., de Klerk, N. H., Davis, E. A., & Jones, T. W. (2016). School performance in children with type 1 diabetes: A contemporary population-based study. Pediatric Diabetes, 17(2), 101–111. https://doi.org/10.1111/pedi.12243

Evans-Cheung, T. C., Bodansky, H. J., Parslow, R. C., & Feltbower, R. G. (2018). Mortality and acute complications in children and young adults diagnosed with Type 1 diabetes in Yorkshire, UK: A cohort study. Diabetic Medicine, 35(1), 112–120. https://doi.org/10.1111/dme.13544

Fleming, M., Fitton, C. A., Steiner, M. F. C., McLay, J. S., Clark, D., King, A., Lindsay, R. S., Mackay, D. F., & Pell, J. P. (2019). Educational and Health Outcomes of Children Treated for Type 1 Diabetes: Scotland-Wide Record Linkage Study of 766,047 Children. Diabetes Care, 42(9), 1700–1707. https://doi.org/10.2337/dc18-2423

Gartner, A., Daniel, R., Farewell, D., Paranjothy, S., Townson, J., & Gregory, J. W. (2020). Demographic and socioeconomic patterns in the risk of alcohol-related hospital admission in children and young adults with childhood onset type-1 diabetes from a record-linked longitudinal population cohort study in Wales. Pediatric Diabetes, 21(7), 1333–1342. https://doi.org/10.1111/pedi.13089

Helgeson, V. S., Palladino, D. K., Reynolds, K. A., Becker, D., Escobar, O., & Siminerio, L. (2014). Early Adolescent Relationship Predictors of Emerging Adult Outcomes: Youth With and Without Type 1 Diabetes. Annals of Behavioral Medicine, 47(3), 270–279. https://doi.org/10.1007/s12160-013-9552-0

Helgeson, V. S., Wright, A., Vaughn, A., Becker, D., & Libman, I. (2022). 14-Year Longitudinal Trajectories of Depressive Symptoms Among Youth With and Without Type 1 Diabetes. Journal of Pediatric Psychology, 47(10), 1135–1144. https://doi.org/10.1093/jpepsy/jsac054

Jacobson, A. m., Hauser, S. t., Cole, C., Willett, J. b., Wolfsdorf, J. i., Dvorak, R., Wolpert, H., Herman, L., & de Groot, M. (1997). Social Relationships Among Young Adults with Insulin-dependent Diabetes Mellitus: Ten-year Follow-up of an Onset Cohort. Diabetic Medicine, 14(1), 73–79. https://doi.org/10.1002/(SICI)1096-9136(199701)14:1<73::AID-DIA294>3.0.CO;2-Q

Laing, S. P., Jones, M. E., Swerdlow, A. J., Burden, A. C., & Gatling, W. (2005). Psychosocial and Socioeconomic Risk Factors for Premature Death in Young People With Type 1 Diabetes. Diabetes Care, 28(7), 1618–1623. https://doi.org/10.2337/diacare.28.7.1618

Lind, T., Waernbaum, I., Berhan, Y., & Dahlquist, G. (2012). Socioeconomic factors, rather than diabetes mellitus per se, contribute to an excessive use of antidepressants among young adults with childhood onset type 1 diabetes mellitus: A register-based study. Diabetologia, 55(3), 617–624. https://doi.org/10.1007/s00125-011-2405-0

Lindkvist, E. B., Thorsen, S. U., Paulsrud, C., Thingholm, P. R., Eriksen, T. L. M., Gaulke, A., Skipper, N., & Svensson, J. (2022). Association of type 1 diabetes and educational achievement in 16–20-year-olds: A Danish nationwide register study. Diabetic Medicine, 39(2), e14673. https://doi.org/10.1111/dme.14673

Liu, S., Leone, M., Ludvigsson, J. F., Lichtenstein, P., D’Onofrio, B., Svensson, A.-M., Gudbjörnsdottir, S., Bergen, S. E., Larsson, H., Kuja-Halkola, R., & Butwicka, A. (2022). Association and Familial Coaggregation of Childhood-Onset Type 1 Diabetes With Depression, Anxiety, and Stress-Related Disorders: A Population-Based Cohort Study. Diabetes Care, 45(9), 1987–1993. https://doi.org/10.2337/dc21-1347

Liu, S., Ludvigsson, J. F., Lichtenstein, P., Gudbjörnsdottir, S., Taylor, M. J., Larsson, H., Kuja-Halkola, R., & Butwicka, A. (2023). Educational Outcomes in Children and Adolescents With Type 1 Diabetes and Psychiatric Disorders. JAMA Network Open, 6(4), e238135. https://doi.org/10.1001/jamanetworkopen.2023.8135

Manderbacka, K., Sund, R., Koski, S., Keskimäki, I., & Elovainio, M. (2011). Diabetes and depression? Secular trends in the use of antidepressants among persons with diabetes in Finland in 1997–2007. Pharmacoepidemiology and Drug Safety, 20(4), 338–343. https://doi.org/10.1002/pds.2072

Matsushima, M., Shimizu, K., Maruyama, M., Nishimura, R., LaPorte, R. E., Tajima, N., & The Diabetes Epidemiology Research International (DERI) US-Japan Mortality Study Group. (1996). Socioeconomic and behavioural risk factors for mortality of individuals with IDDM in Japan: Population-based case-control study. Diabetologia, 39(6), 710–716. https://doi.org/10.1007/BF00418543

Matsushima, M., Tajima, N., Agata, T., Yokoyama, J., Ikeda, Y., & Isogai, Y. (1993). Social and Economic Impact on Youth-Onset Diabetes in Japan. Diabetes Care, 16(5), 824–827. https://doi.org/10.2337/diacare.16.5.824

McGrady, M. E., & Hood, K. K. (2010). Depressive symptoms in adolescents with type 1 diabetes: Associations with longitudinal outcomes. Diabetes Research and Clinical Practice, 88(3), e35–e37. https://doi.org/10.1016/j.diabres.2010.03.025

Meo, S. A., Alkahlan, M. A., Al-mubarak, M. A., Al-obayli, M. S., Melaibary, B. A., Dous, A. N. B., & Alhassoun, A. I. (2013). Impact of type 1 diabetes mellitus on academic performance. Journal of International Medical Research, 41(3), 855–858. https://doi.org/10.1177/0300060513483417

Milton, B., Holland, P., & Whitehead, M. (2006). The social and economic consequences of childhood-onset Type 1 diabetes mellitus across the lifecourse: A systematic review. Diabetic Medicine, 23(8), 821–829. https://doi.org/10.1111/j.1464-5491.2006.01796.x

Mitchell, R. J., McMaugh, A., Woodhead, H., Lystad, R. P., Zurynski, Y., Badgery-Parker, T., Cameron, C. M., & Hng, T.-M. (2022). The impact of type 1 diabetes mellitus in childhood on academic performance: A matched population-based cohort study. Pediatric Diabetes, 23(3), 411–420. https://doi.org/10.1111/pedi.13317

Plener, P. L., Molz, E., Berger, G., Schober, E., Mönkemöller, K., Denzer, C., Goldbeck, L., & Holl, R. W. (2015). Depression, metabolic control, and antidepressant medication in young patients with type 1 diabetes. Pediatric Diabetes, 16(1), 58–66. https://doi.org/10.1111/pedi.12130

Secrest, A. M., Costacou, T., Gutelius, B., Miller, R. G., Songer, T. J., & Orchard, T. J. (2011). Association of Socioeconomic Status with Mortality in Type 1 Diabetes: The Pittsburgh Epidemiology of Diabetes Complications Study. Annals of Epidemiology, 21(5), 367–373. https://doi.org/10.1016/j.annepidem.2011.02.011

Sjöberg, L., Pitkäniemi, J., Haapala, L., Kaaja, R., & Tuomilehto, J. (2013). Fertility in people with childhood-onset type 1 diabetes. Diabetologia, 56(1), 78–81. https://doi.org/10.1007/s00125-012-2731-x

Skipper, N., Gaulke, A., Sildorf, S. M., Eriksen, T. M., Nielsen, N. F., & Svensson, J. (2019). Association of Type 1 Diabetes With Standardized Test Scores of Danish Schoolchildren. JAMA, 321(5), 484–492. https://doi.org/10.1001/jama.2018.21819

Steen Carlsson, K., Landin-Olsson, M., Nyström, L., Arnqvist, H. J., Bolinder, J., Östman, J., & Gudbjörnsdóttir, S. (2010). Long-term detrimental consequences of the onset of type 1 diabetes on annual earnings—Evidence from annual registry data in 1990–2005. Diabetologia, 53(6), 1084–1092. https://doi.org/10.1007/s00125-009-1625-z

Thorsted, A. B., Thygesen, L. C., Hoffmann, S. H., Rosenkilde, S., Lehn, S. F., Lundby-Christensen, L., & Horsbøl, T. A. (2024). Educational outcomes and the role of comorbidity among adolescents with type 1-diabetes in Denmark. Diabetic Medicine, 41(5), e15270. <https://doi.org/10.1111/dme.15270>

## Supplementary figures


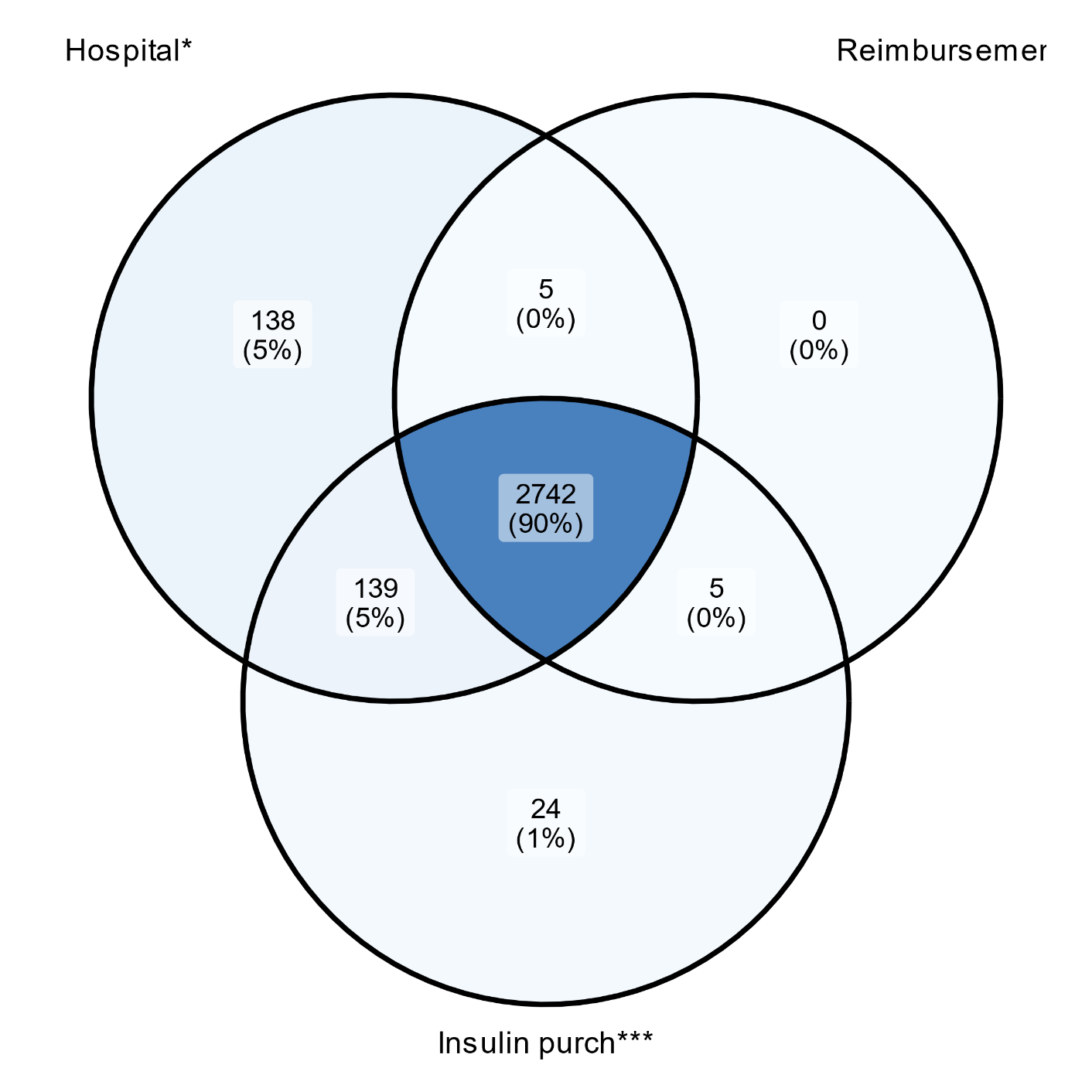


Supplementary Figure 1. Identification of type 1 diabetes from the tree register sources. *Hospital= Hospital Discharge Register/ Care Register for Health Care, Reimbursement= The Register of Special Reimbursement of medicines. ***Insulin purch= Prescription database (1994-2017). Note that 5 means 5 or less than 5.


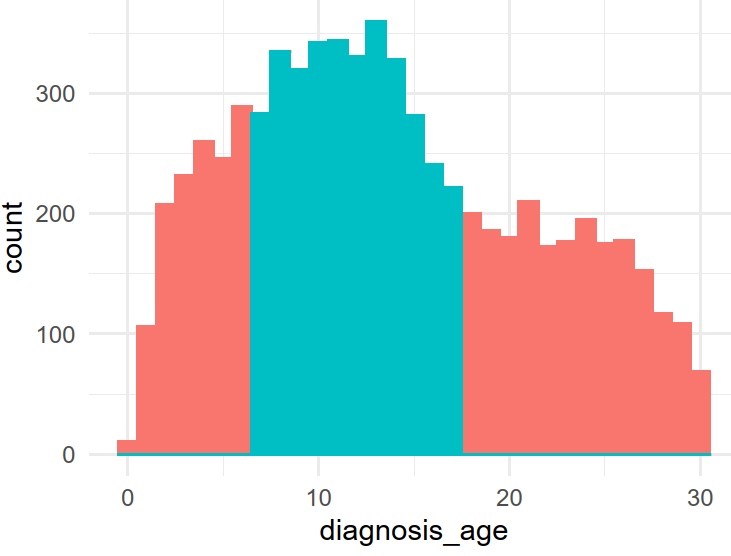


Supplementary Figure 2. Distribution of Type 1 diabetes diagnosis age in the population. Blue are is the exposed in the current study.


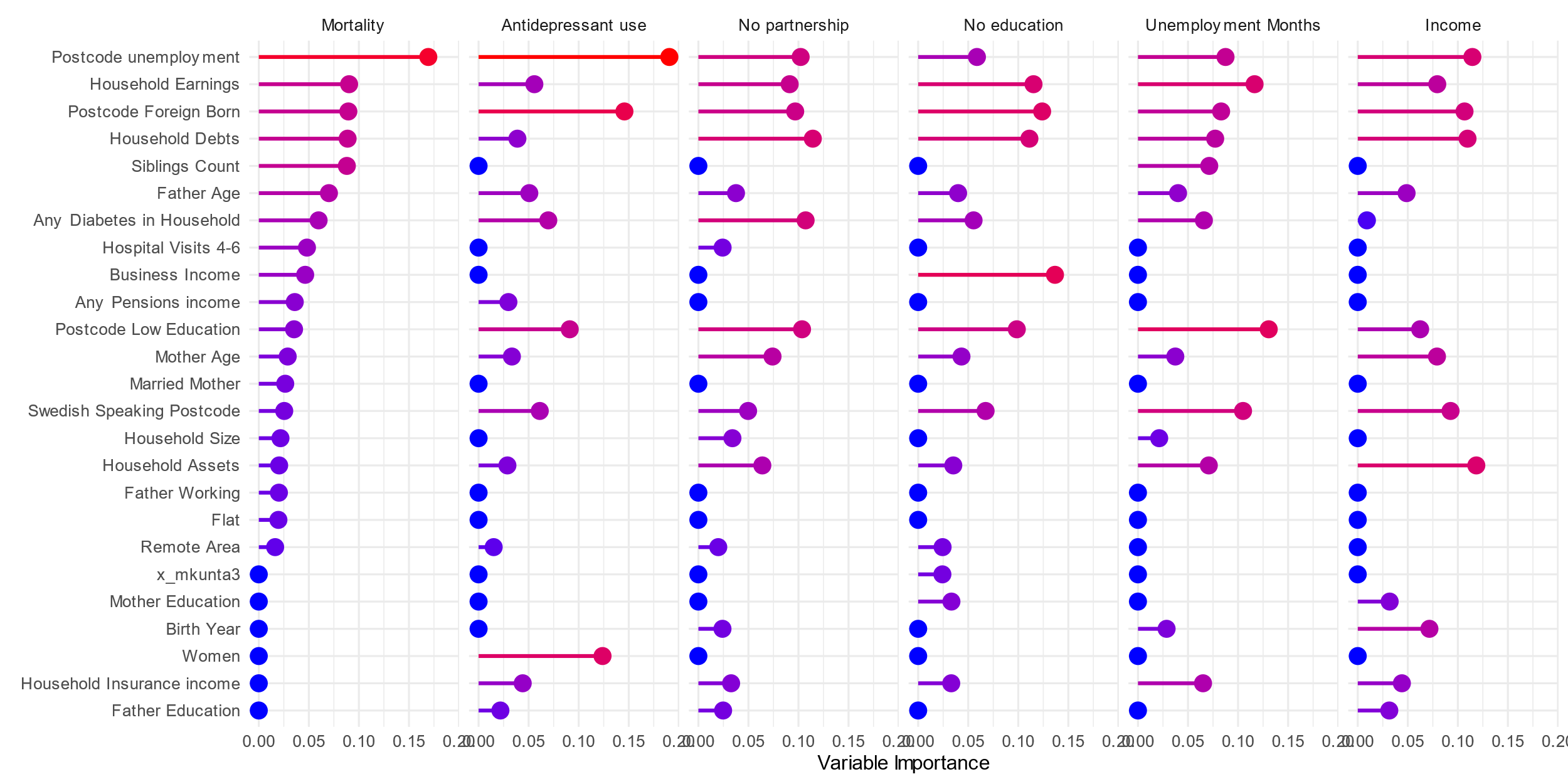
Supplementary Figure 3. Variable importance values in Causal Forest models by outcomes. A higher value indicates a higher (weighted) number of times variable was used to make split in estimating the conditional average treatment effects. Zero value indicates that the covariate was not used in the final forest due to low signal in the pilot forest. Finnish register study.


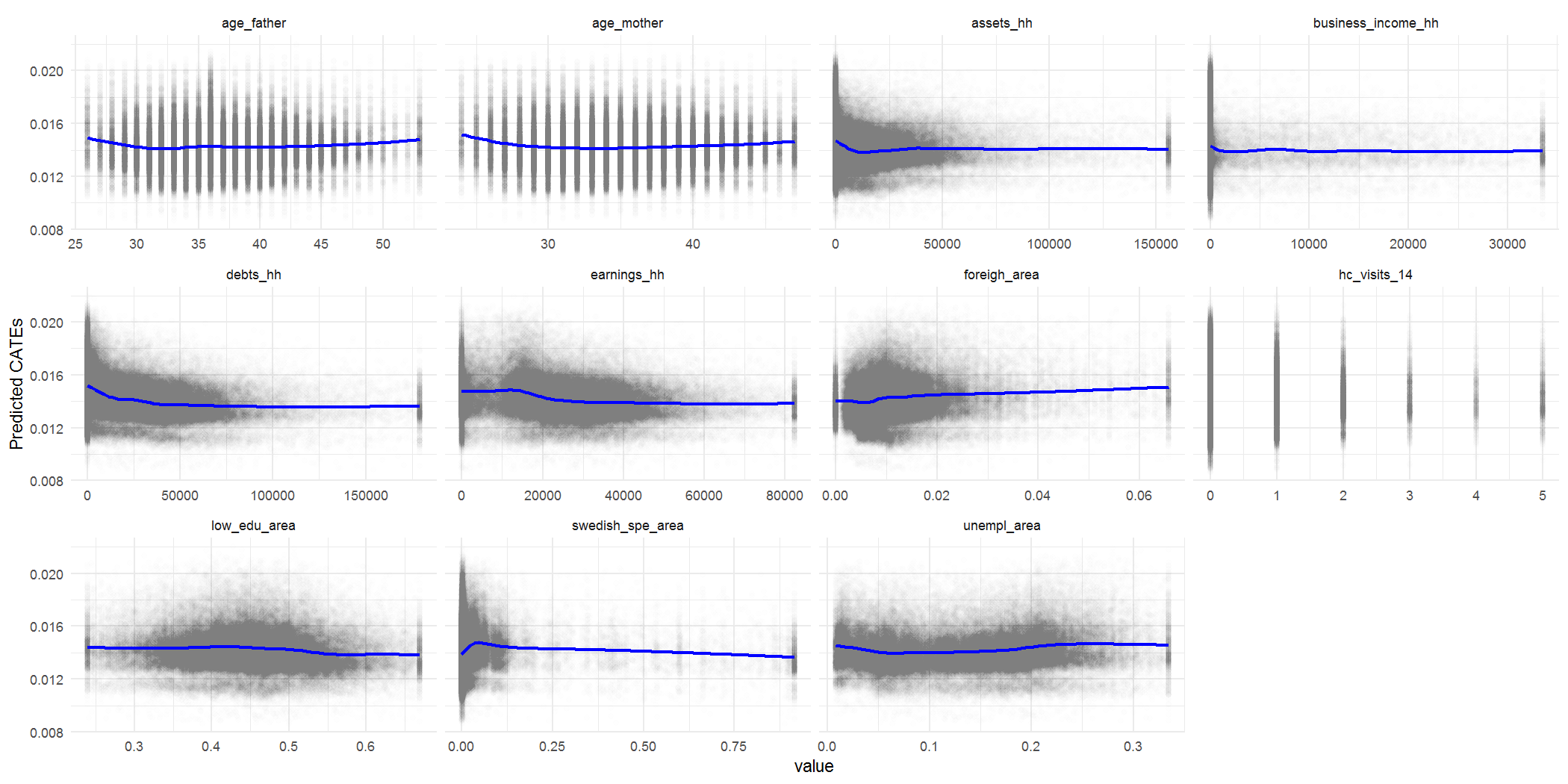


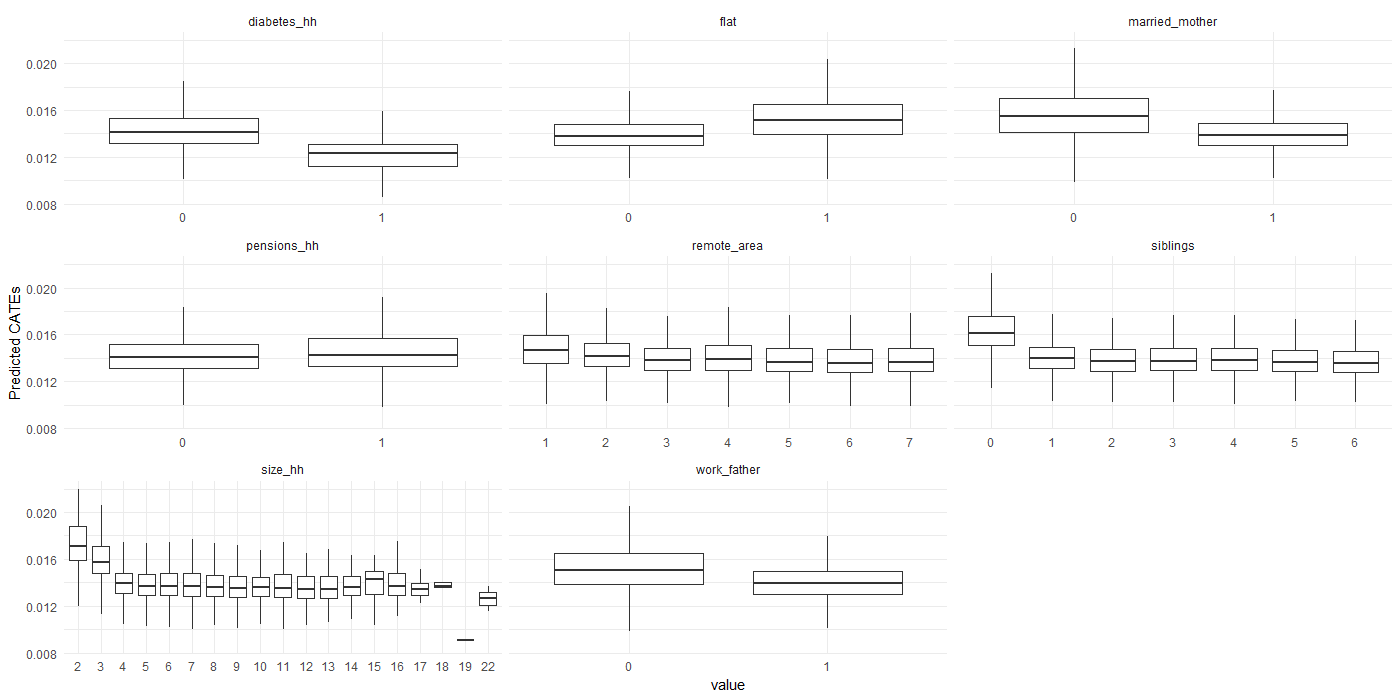


Supplementary Figure 4. The relationships between covariates and estimated conditional average treatment effects of T1D on mortality. Scatter plot with smoothed fitted line is provide for continuous variables. All covariates bottom and top coded to 1^st^ and 99^th^ percent and jitter added. Finnish register study.


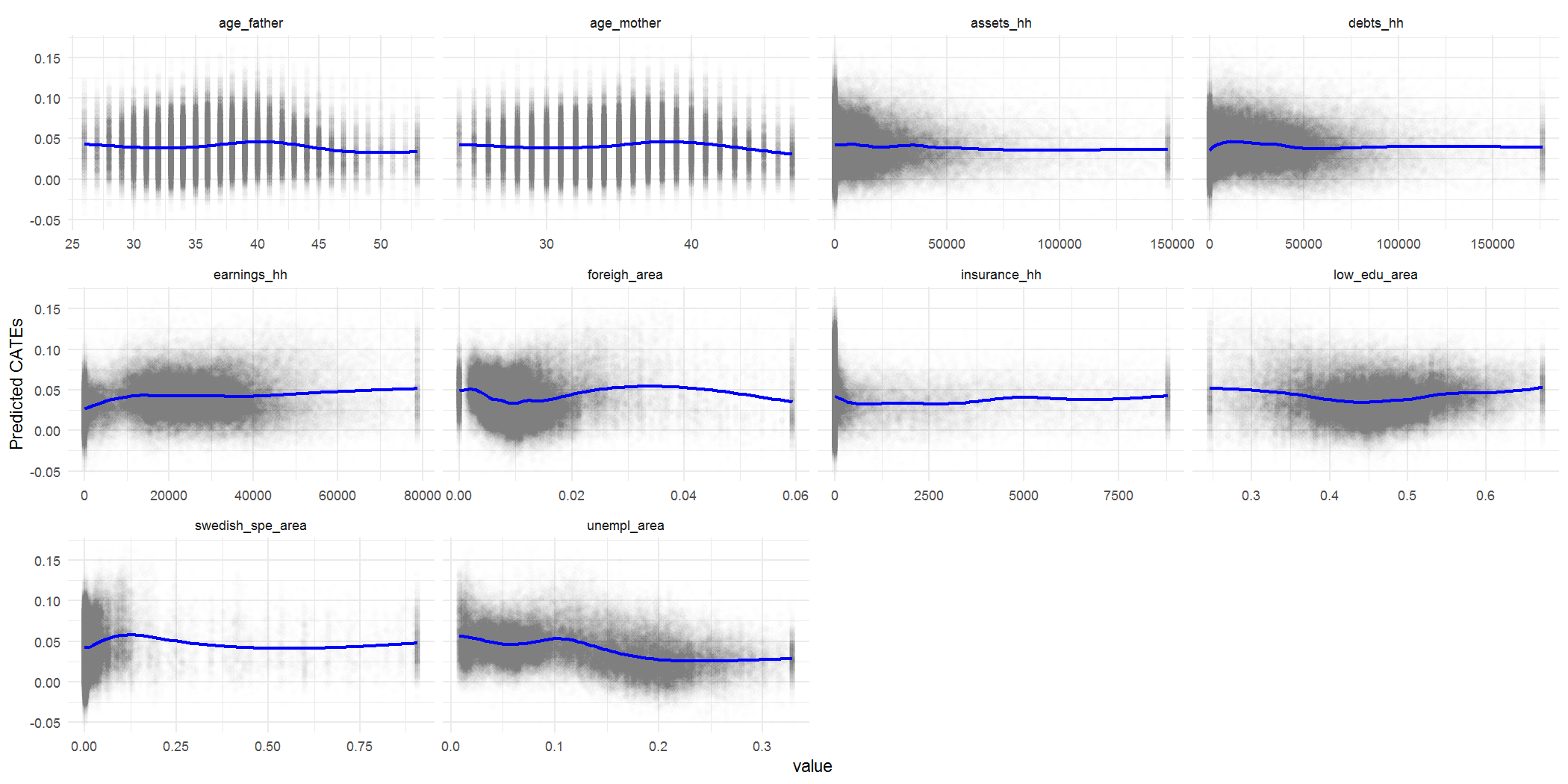

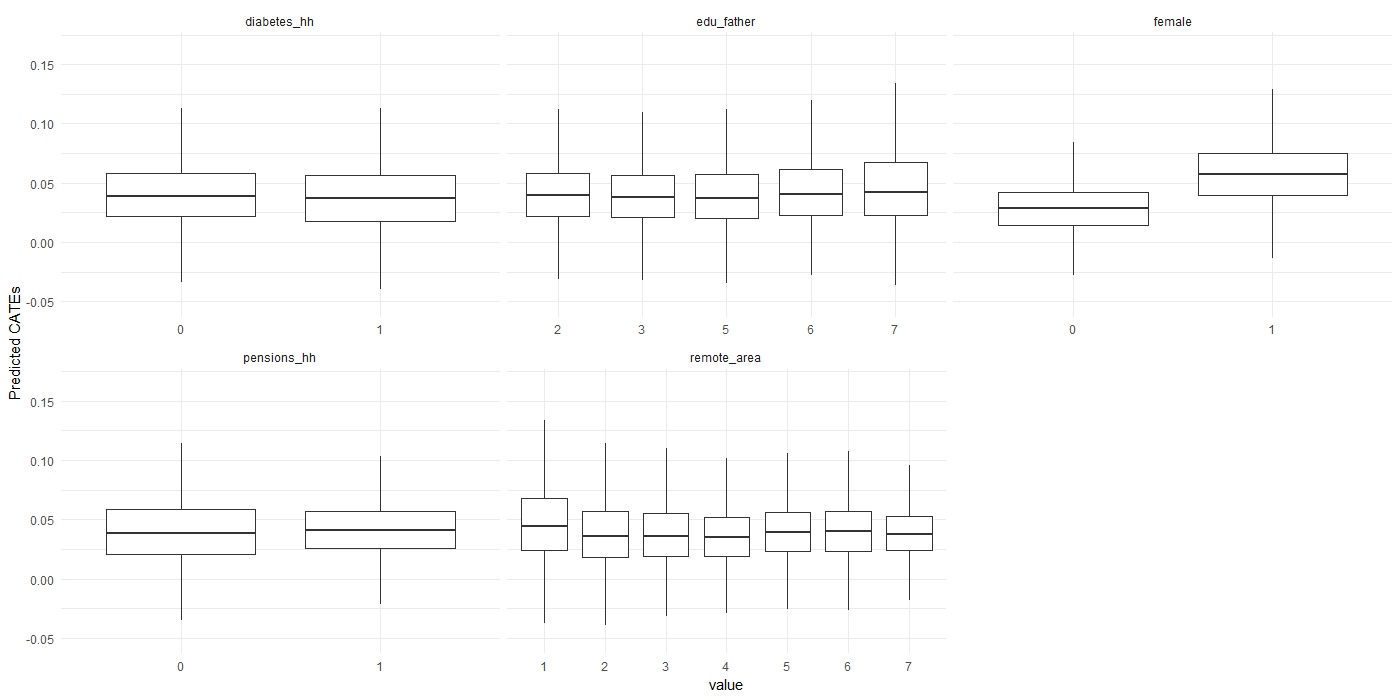


Supplementary Figure 5. The relationships between covariates and estimated conditional average treatment effects of T1D on depression medication. Scatter plot with smoothed fitted line is provide for continuous variables. Finnish register study. All covariates bottom and top coded to 1^st^ and 99^th^ percent and jitter added. Finnish register study.


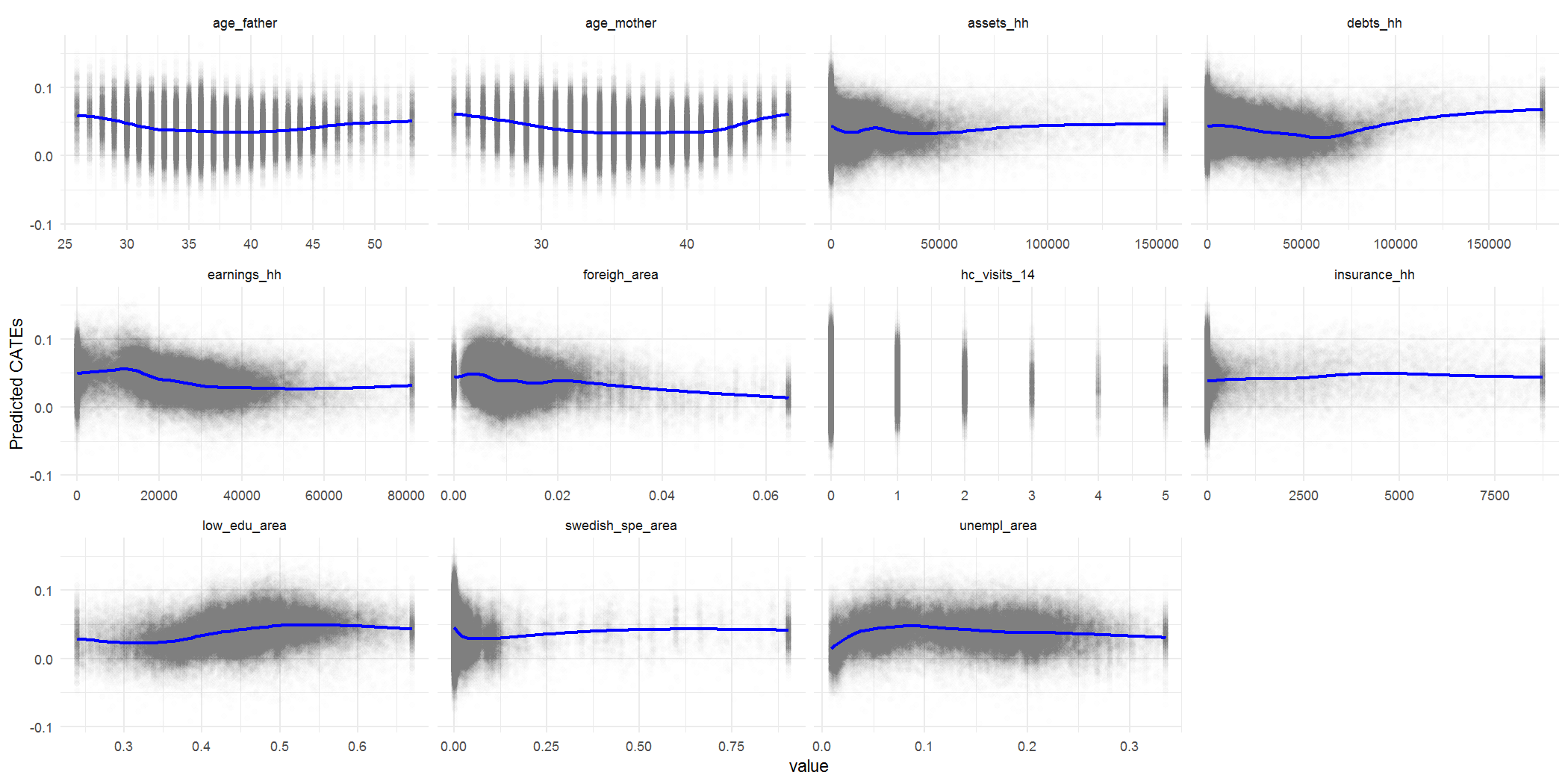

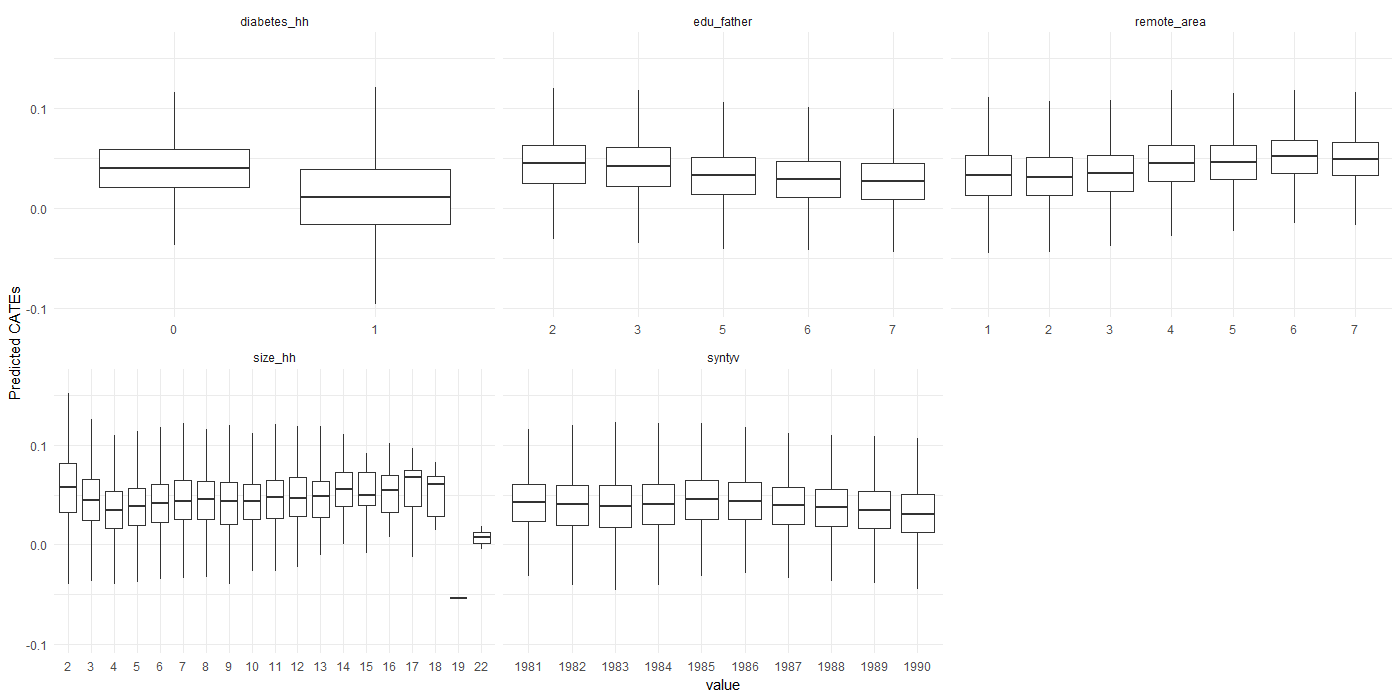


Supplementary Figure 6. The relationships between covariates and estimated conditional average treatment effects of T1D on partnership. Scatter plot with smoothed fitted line is provide for continuous variables. Finnish register study. All covariates bottom and top coded to 1^st^ and 99^th^ percent and jitter added. Finnish register study.


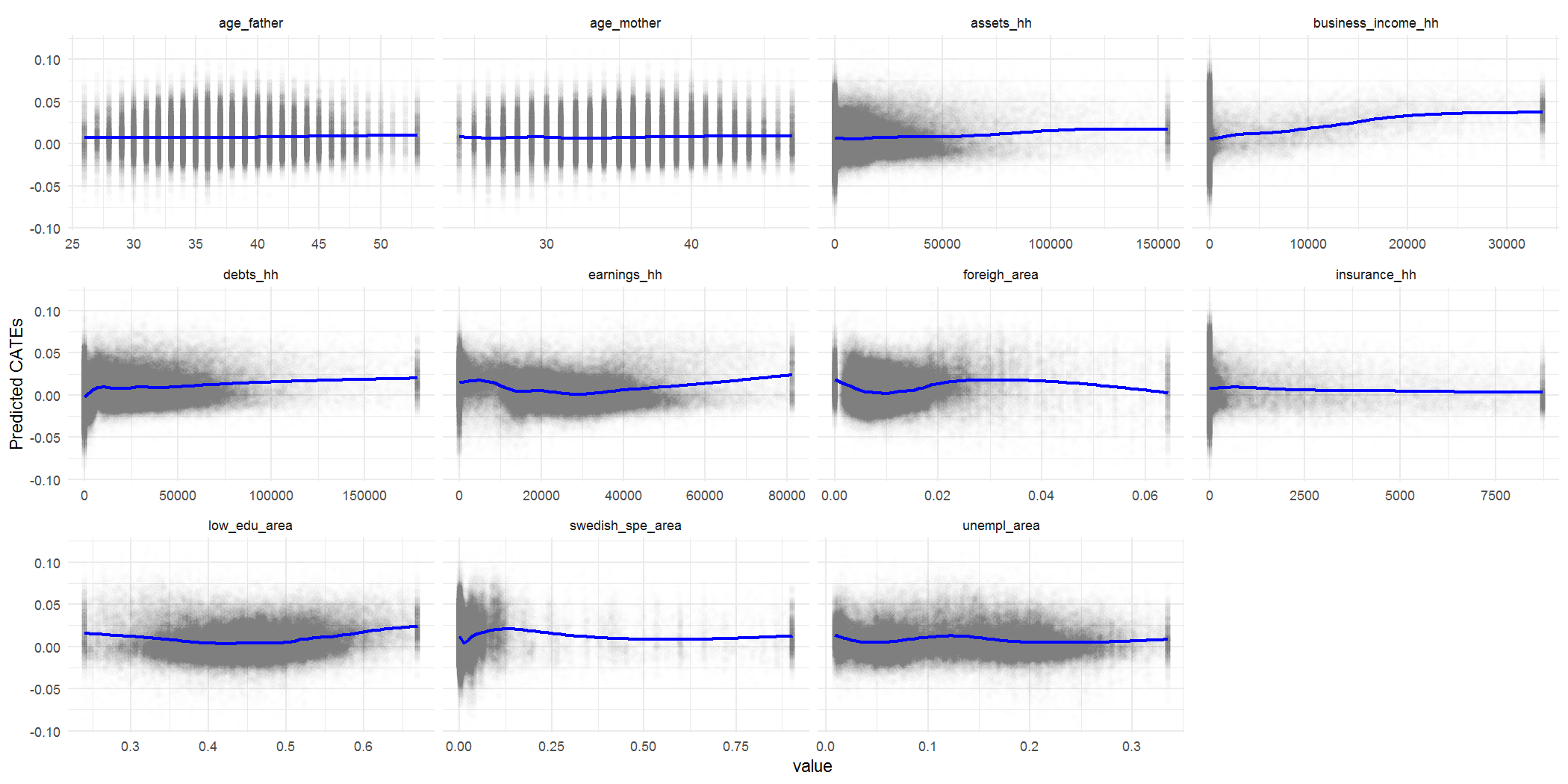

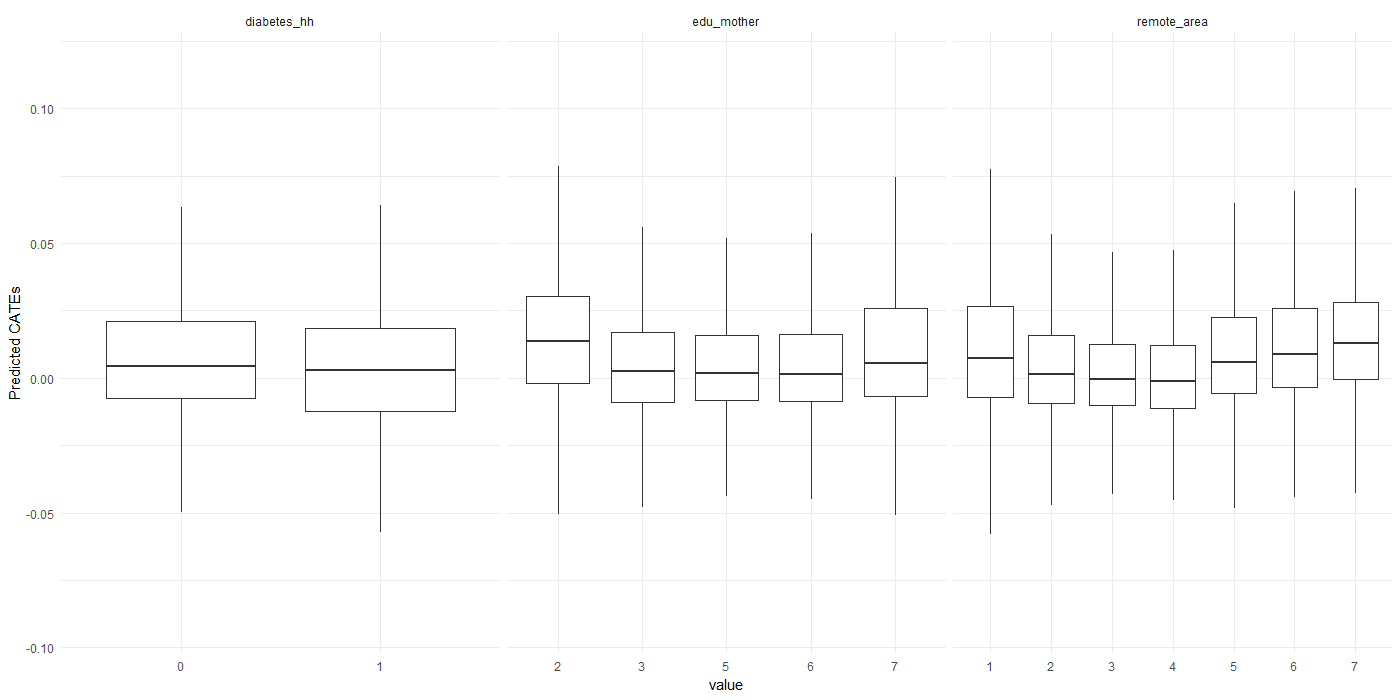


Supplementary Figure 7. The relationships between covariates and estimated conditional average treatment effects of T1D on education. Scatter plot with smoothed fitted line is provide for continuous variables. Finnish register study. All covariates bottom and top coded to 1^st^ and 99^th^ percent and jitter added. Finnish register study.


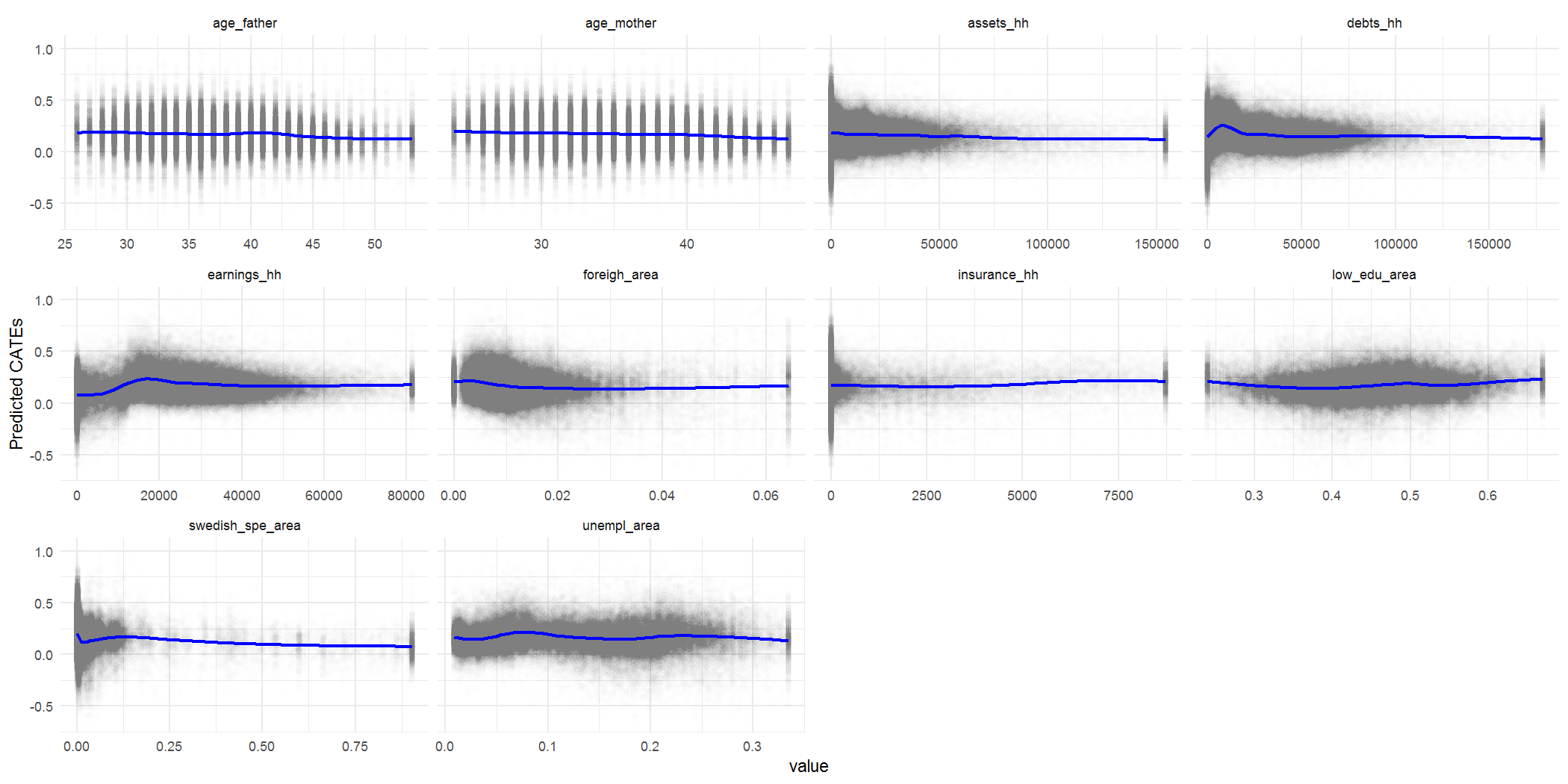

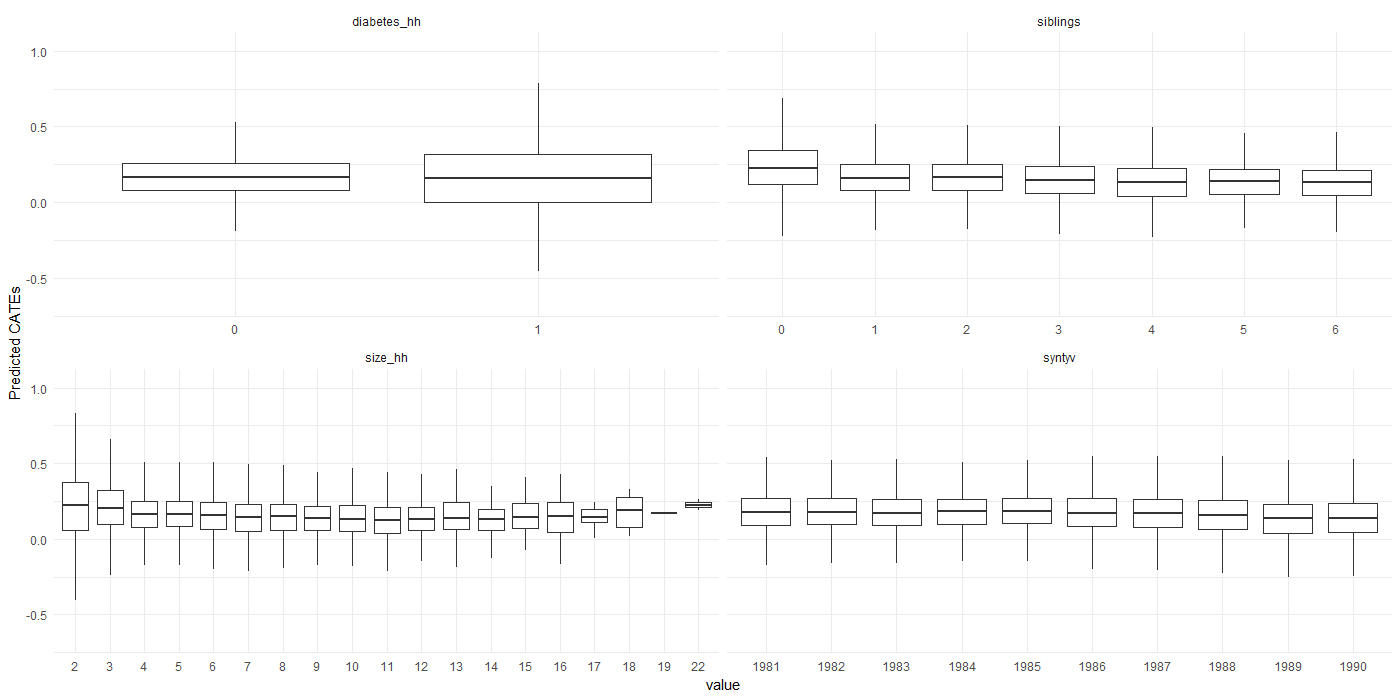


Supplementary Figure 8. The relationships between covariates and estimated conditional average treatment effects of T1D on unemployment months. Scatter plot with smoothed fitted line is provide for continuous variables. Finnish register study. All covariates bottom and top coded to 1^st^ and 99^th^ percent and jitter added. Finnish register study.


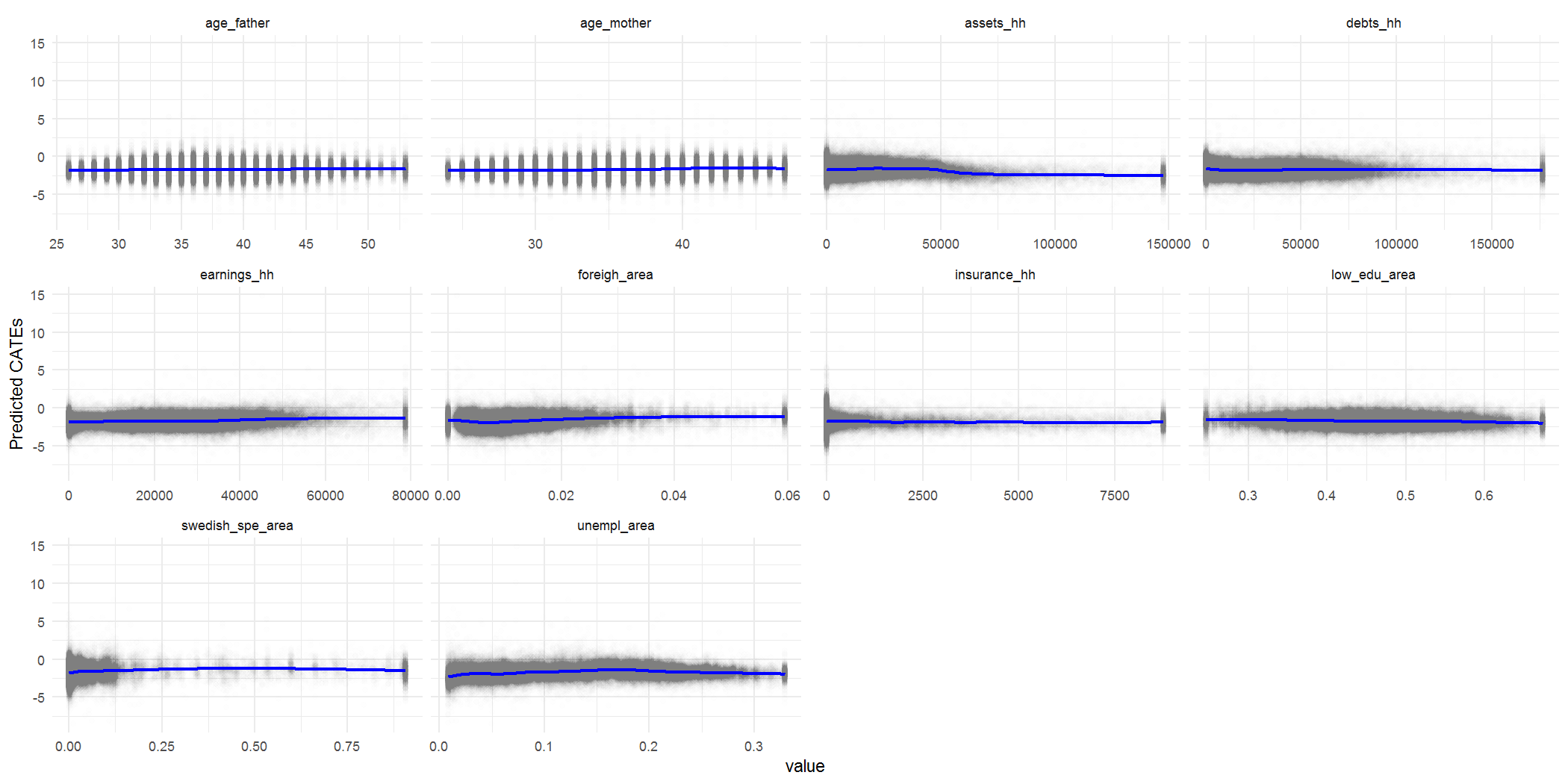

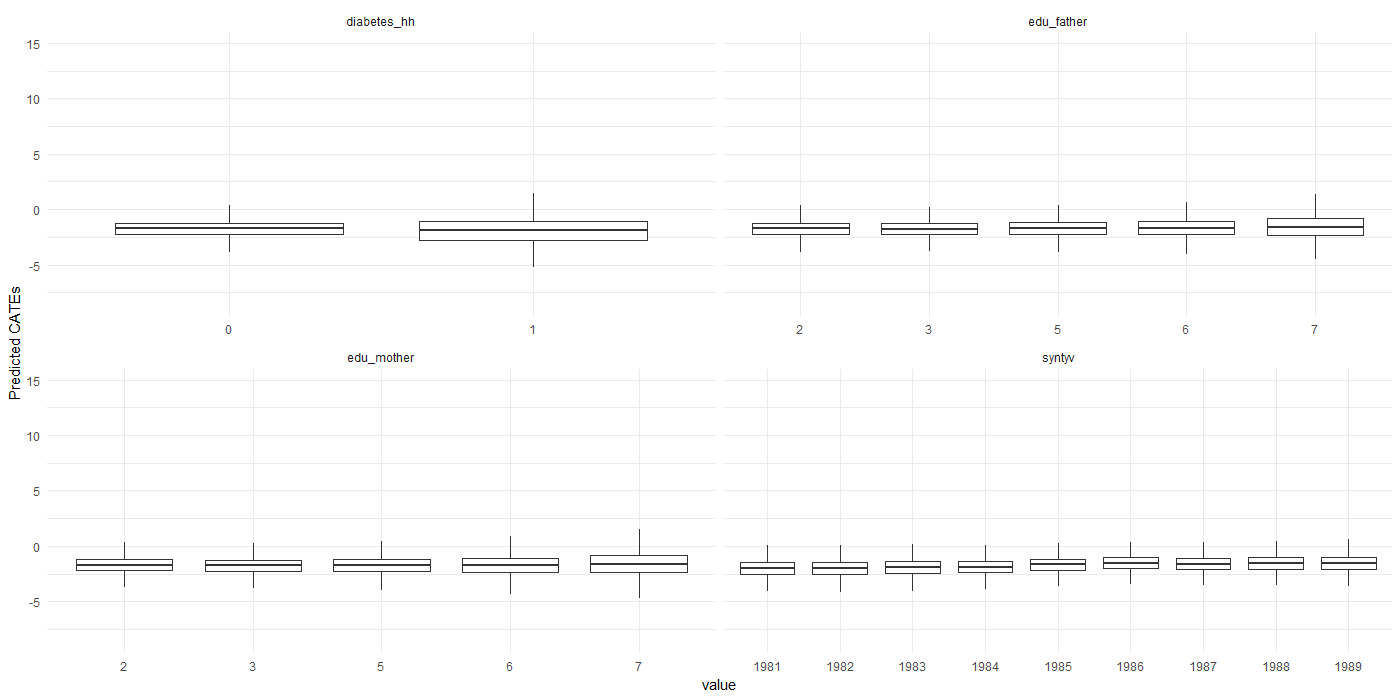


Supplementary Figure 9. The relationships between covariates and estimated conditional average treatment effects of T1D on income. Scatter plot with smoothed fitted line is provide for continuous variables. Finnish register study. All covariates bottom and top coded to 1^st^ and 99^th^ percent and jitter added. Finnish register study.

## Supplementary tables

Supplementary table 1. Characteristics of the Type 1 diabetes (T1D) study population, matched sample and full population. Finnish register study. Means/fractions.

| term | Matched group | T1D group | Full population |
| --- | --- | --- | --- |
| number_obs | 78,883 | 3,048 | 626,931 |
| Any Diabetes in Household | 0.02 | 0.11 | 0.03 |
| Any Home Care | 0.23 | 0.21 | 0.23 |
| Any Pensions income | 0.09 | 0.09 | 0.09 |
| Any Social Assistance | 0.12 | 0.10 | 0.11 |
| Any Unemployment Benefits | 0.33 | 0.33 | 0.32 |
| Birth Year | 1,985.75 | 1,985.75 | 1,985.46 |
| Business Income | 1,882.95 | 1,927.04 | 1,839.84 |
| cohort 1981 | 0.09 | 0.09 | 0.10 |
| cohort 1982 | 0.08 | 0.08 | 0.10 |
| cohort 1983 | 0.09 | 0.09 | 0.10 |
| cohort 1984 | 0.10 | 0.10 | 0.10 |
| cohort 1985 | 0.10 | 0.10 | 0.10 |
| cohort 1986 | 0.10 | 0.10 | 0.10 |
| cohort 1987 | 0.11 | 0.11 | 0.09 |
| cohort 1988 | 0.10 | 0.10 | 0.10 |
| cohort 1989 | 0.11 | 0.11 | 0.10 |
| cohort 1990 | 0.12 | 0.12 | 0.10 |
| Crowded Household | 0.50 | 0.50 | 0.49 |
| Father Age | 36.99 | 37.19 | 36.96 |
| Father edu level 1 | 0.29 | 0.27 | 0.29 |
| Father edu level 2 | 0.42 | 0.43 | 0.42 |
| Father edu level 3 | 0.13 | 0.14 | 0.13 |
| Father edu level 4 | 0.07 | 0.07 | 0.07 |
| Father edu level 5 | 0.09 | 0.08 | 0.09 |
| Father Education | 3.54 | 3.55 | 3.54 |
| Father Health edu | 0.03 | 0.03 | 0.03 |
| Father hospital Visits | 0.28 | 0.34 | 0.39 |
| Father Working | 0.83 | 0.83 | 0.84 |
| Flat | 0.25 | 0.26 | 0.25 |
| Home Owner | 0.75 | 0.75 | 0.76 |
| Hospital Visits 4-6 | 0.39 | 0.47 | 0.51 |
| Household Assets | 21,699.77 | 20,445.39 | 21,237.23 |
| Household Debts | 33,898.69 | 32,984.45 | 33,838.30 |
| Household Earnings | 26,571.45 | 25,680.74 | 26,300.09 |
| Household Insurance income | 609.60 | 629.43 | 609.78 |
| Household Size | 4.55 | 4.47 | 4.55 |
| Married Mother | 0.82 | 0.82 | 0.82 |
| Missing Father | 0.02 | 0.02 | 0.02 |
| Mother Age | 34.67 | 34.83 | 34.61 |
| Mother edu level 1 | 0.23 | 0.23 | 0.23 |
| Mother edu level 2 | 0.45 | 0.46 | 0.45 |
| Mother edu level 3 | 0.20 | 0.20 | 0.20 |
| Mother edu level 4 | 0.06 | 0.05 | 0.06 |
| Mother edu level 5 | 0.06 | 0.06 | 0.06 |
| Mother Education | 3.59 | 3.57 | 3.58 |
| Mother Health edu | 0.17 | 0.17 | 0.17 |
| Mother Hospital Visits | 1.00 | 1.02 | 1.00 |
| Mother Working | 0.71 | 0.71 | 0.71 |
| Postcode Foreign Born | 0.01 | 0.01 | 0.01 |
| Postcode Low Education | 0.45 | 0.45 | 0.45 |
| Postcode unemployment | 0.14 | 0.14 | 0.13 |
| Remote Area | 3.29 | 3.28 | 3.30 |
| Siblings Count | 1.49 | 1.43 | 1.49 |
| Single Parent | 0.12 | 0.11 | 0.11 |
| Swedish Speaking | 0.05 | 0.04 | 0.05 |
| Swedish Speaking Postcode | 0.05 | 0.05 | 0.06 |
| Women | 0.42 | 0.43 | 0.49 |

Supplementary table 2. The means (standard deviations) of covariates at each ranked CATE fourth for Mortality. Observations assigned to CATE groups using Causal Forest.

| Variable | 1st CATE Rank | 2nd | 3rd | 4th CATE Rank |
| --- | --- | --- | --- | --- |
| Any Diabetes in Household | 0.10 (0.29) | 0.01 (0.09) | 0.01 (0.08) | 0.00 (0.05) |
| Any Home Care | 0.28 (0.45) | 0.27 (0.44) | 0.25 (0.43) | 0.12 (0.33) |
| Any Pensions income | 0.06 (0.24) | 0.07 (0.25) | 0.10 (0.30) | 0.12 (0.32) |
| Any Social Assistance | 0.06 (0.24) | 0.07 (0.25) | 0.12 (0.32) | 0.22 (0.41) |
| Any Unemployment Benefits | 0.26 (0.44) | 0.29 (0.45) | 0.35 (0.48) | 0.41 (0.49) |
| Birth Year | 1985.45 (2.80) | 1985.70 (2.87) | 1985.82 (2.89) | 1986.02 (2.87) |
| Business Income | 2038.41 (6953.55) | 2431.05 (8167.43) | 2165.35 (8874.35) | 903.54 (4699.24) |
| Crowded Household | 0.51 (0.50) | 0.52 (0.50) | 0.57 (0.49) | 0.40 (0.49) |
| Father Age | 36.33 (5.17) | 37.11 (5.23) | 37.52 (5.65) | 37.03 (6.36) |
| Father Education | 3.78 (1.67) | 3.75 (1.66) | 3.51 (1.57) | 3.12 (1.38) |
| Father Health edu | 0.03 (0.17) | 0.03 (0.17) | 0.02 (0.15) | 0.02 (0.12) |
| Father Working | 0.95 (0.22) | 0.92 (0.27) | 0.83 (0.38) | 0.63 (0.48) |
| Father hospital Visits | 0.24 (0.81) | 0.24 (0.79) | 0.28 (1.01) | 0.39 (1.41) |
| Flat | 0.05 (0.22) | 0.12 (0.33) | 0.28 (0.45) | 0.54 (0.50) |
| Home Owner | 0.90 (0.30) | 0.86 (0.34) | 0.75 (0.43) | 0.50 (0.50) |
| Hospital Visits 4-6 | 0.32 (1.27) | 0.38 (1.31) | 0.42 (1.64) | 0.46 (1.57) |
| Household Assets | 21438.22 (54367.53) | 25325.95 (61448.53) | 26106.24 (85670.56) | 13742.67 (42263.29) |
| Household Debts | 51406.18 (53485.35) | 41593.05 (58156.59) | 26988.07 (38254.72) | 15465.98 (26385.65) |
| Household Earnings | 31610.60 (57672.48) | 30703.88 (151284.58) | 25325.53 (106565.25) | 18511.86 (15977.50) |
| Household Insurance income | 742.72 (1959.38) | 687.40 (1887.56) | 665.24 (1878.36) | 345.96 (1298.34) |
| Household Size | 5.04 (1.51) | 4.90 (1.42) | 4.71 (1.35) | 3.54 (1.25) |
| Married Mother | 0.97 (0.16) | 0.95 (0.22) | 0.86 (0.35) | 0.49 (0.50) |
| Missing Father | 0.00 (0.05) | 0.00 (0.06) | 0.01 (0.11) | 0.08 (0.26) |
| Mother Age | 34.23 (4.81) | 34.93 (4.84) | 35.13 (5.16) | 34.41 (5.83) |
| Mother Education | 3.78 (1.51) | 3.76 (1.51) | 3.54 (1.46) | 3.28 (1.36) |
| Mother Health edu | 0.20 (0.40) | 0.19 (0.39) | 0.16 (0.36) | 0.12 (0.32) |
| Mother Hospital Visits | 1.07 (1.15) | 1.02 (1.16) | 1.01 (1.17) | 0.90 (1.38) |
| Mother Working | 0.76 (0.42) | 0.74 (0.44) | 0.68 (0.47) | 0.65 (0.48) |
| Postcode Foreign Born | 0.01 (0.01) | 0.01 (0.01) | 0.01 (0.01) | 0.02 (0.01) |
| Postcode Low Education | 0.46 (0.10) | 0.45 (0.09) | 0.45 (0.09) | 0.44 (0.08) |
| Postcode unemployment | 0.13 (0.07) | 0.13 (0.08) | 0.14 (0.09) | 0.15 (0.09) |
| Remote Area | 4.07 (1.98) | 3.42 (1.99) | 3.10 (2.04) | 2.56 (1.86) |
| Siblings Count | 1.84 (1.09) | 1.74 (1.05) | 1.61 (1.03) | 0.78 (0.98) |
| Single Parent | 0.01 (0.10) | 0.02 (0.14) | 0.07 (0.25) | 0.38 (0.49) |
| Swedish Speaking | 0.06 (0.24) | 0.06 (0.23) | 0.04 (0.20) | 0.03 (0.18) |
| Swedish Speaking Postcode | 0.06 (0.19) | 0.06 (0.17) | 0.05 (0.14) | 0.04 (0.12) |
| Women | 0.43 (0.50) | 0.42 (0.49) | 0.42 (0.49) | 0.42 (0.49) |

Supplementary table 3. The means (standard deviations) of covariates at each ranked CATE fourth for Antidepressant use. Observations assigned to CATE groups using Causal Forest.

| Variable | 1st CATE Rank | 2nd | 3rd | 4th CATE Rank |
| --- | --- | --- | --- | --- |
| Any Diabetes in Household | 0.03 (0.18) | 0.03 (0.16) | 0.03 (0.17) | 0.02 (0.16) |
| Any Home Care | 0.26 (0.44) | 0.24 (0.43) | 0.23 (0.42) | 0.19 (0.39) |
| Any Pensions income | 0.06 (0.24) | 0.08 (0.28) | 0.10 (0.30) | 0.07 (0.25) |
| Any Social Assistance | 0.15 (0.35) | 0.11 (0.31) | 0.10 (0.30) | 0.08 (0.27) |
| Any Unemployment Benefits | 0.40 (0.49) | 0.33 (0.47) | 0.29 (0.46) | 0.24 (0.43) |
| Birth Year | 1986.38 (2.25) | 1985.16 (2.54) | 1984.67 (2.53) | 1984.49 (2.47) |
| Business Income | 2267.41 (7906.57) | 1992.25 (7094.46) | 1908.76 (6730.52) | 1191.28 (7558.19) |
| Crowded Household | 0.52 (0.50) | 0.50 (0.50) | 0.48 (0.50) | 0.47 (0.50) |
| Father Age | 36.90 (6.07) | 37.11 (5.65) | 36.91 (5.43) | 36.78 (5.32) |
| Father Education | 3.51 (1.56) | 3.48 (1.54) | 3.42 (1.53) | 3.65 (1.69) |
| Father Health edu | 0.03 (0.16) | 0.02 (0.15) | 0.02 (0.15) | 0.03 (0.16) |
| Father Working | 0.79 (0.41) | 0.83 (0.37) | 0.85 (0.36) | 0.87 (0.33) |
| Father hospital Visits | 0.34 (1.07) | 0.28 (1.00) | 0.24 (0.97) | 0.21 (0.86) |
| Flat | 0.24 (0.43) | 0.23 (0.42) | 0.22 (0.42) | 0.29 (0.45) |
| Home Owner | 0.75 (0.44) | 0.77 (0.42) | 0.77 (0.42) | 0.75 (0.43) |
| Hospital Visits 4-6 | 0.45 (1.52) | 0.40 (1.52) | 0.37 (1.40) | 0.31 (1.39) |
| Household Assets | 22217.10 (49680.44) | 21468.32 (48049.36) | 20845.68 (56265.15) | 17619.06 (49648.02) |
| Household Debts | 35243.54 (51543.48) | 33520.27 (47854.69) | 33672.31 (42351.08) | 32685.89 (40794.45) |
| Household Earnings | 24094.33 (18547.82) | 25489.04 (126036.68) | 24148.86 (16511.68) | 31152.64 (165874.38) |
| Household Insurance income | 621.78 (1690.68) | 649.26 (1796.62) | 722.88 (1925.90) | 477.26 (1727.04) |
| Household Size | 4.58 (1.55) | 4.60 (1.55) | 4.59 (1.53) | 4.42 (1.39) |
| Married Mother | 0.81 (0.39) | 0.83 (0.38) | 0.83 (0.37) | 0.83 (0.38) |
| Missing Father | 0.02 (0.15) | 0.02 (0.14) | 0.02 (0.16) | 0.02 (0.15) |
| Mother Age | 34.50 (5.45) | 34.70 (5.21) | 34.54 (5.09) | 34.64 (5.01) |
| Mother Education | 3.56 (1.44) | 3.53 (1.43) | 3.51 (1.44) | 3.63 (1.54) |
| Mother Health edu | 0.17 (0.37) | 0.16 (0.37) | 0.16 (0.37) | 0.17 (0.38) |
| Mother Hospital Visits | 1.03 (1.26) | 1.01 (1.23) | 1.02 (1.16) | 0.94 (1.05) |
| Mother Working | 0.65 (0.48) | 0.70 (0.46) | 0.73 (0.45) | 0.78 (0.42) |
| Postcode Foreign Born | 0.01 (0.01) | 0.01 (0.01) | 0.01 (0.01) | 0.01 (0.01) |
| Postcode Low Education | 0.44 (0.08) | 0.46 (0.09) | 0.47 (0.09) | 0.45 (0.10) |
| Postcode unemployment | 0.17 (0.08) | 0.14 (0.09) | 0.12 (0.08) | 0.10 (0.07) |
| Remote Area | 3.24 (1.94) | 3.46 (2.07) | 3.55 (2.10) | 3.01 (2.03) |
| Siblings Count | 1.53 (1.16) | 1.52 (1.15) | 1.51 (1.13) | 1.39 (1.03) |
| Single Parent | 0.12 (0.33) | 0.11 (0.31) | 0.11 (0.31) | 0.11 (0.32) |
| Swedish Speaking | 0.04 (0.19) | 0.04 (0.20) | 0.04 (0.20) | 0.06 (0.24) |
| Swedish Speaking Postcode | 0.05 (0.14) | 0.05 (0.15) | 0.05 (0.15) | 0.07 (0.17) |
| Women | 0.15 (0.36) | 0.25 (0.43) | 0.47 (0.50) | 0.82 (0.38) |

Supplementary table 4. The means (standard deviations) of covariates at each ranked CATE fourth for No partnership. Observations assigned to CATE groups using Causal Forest.

| Variable | 1st CATE Rank | 2nd | 3rd | 4th CATE Rank |
| --- | --- | --- | --- | --- |
| Any Diabetes in Household | 0.07 (0.25) | 0.02 (0.13) | 0.01 (0.11) | 0.02 (0.13) |
| Any Home Care | 0.22 (0.41) | 0.21 (0.41) | 0.23 (0.42) | 0.26 (0.44) |
| Any Pensions income | 0.07 (0.26) | 0.07 (0.26) | 0.09 (0.29) | 0.11 (0.31) |
| Any Social Assistance | 0.09 (0.29) | 0.10 (0.29) | 0.12 (0.32) | 0.16 (0.37) |
| Any Unemployment Benefits | 0.29 (0.45) | 0.32 (0.47) | 0.35 (0.48) | 0.36 (0.48) |
| Birth Year | 1986.06 (2.98) | 1985.92 (2.87) | 1985.63 (2.83) | 1985.36 (2.73) |
| Business Income | 1110.83 (5505.60) | 1490.44 (6013.55) | 2082.08 (8713.22) | 2923.84 (8701.35) |
| Crowded Household | 0.49 (0.50) | 0.49 (0.50) | 0.51 (0.50) | 0.52 (0.50) |
| Father Age | 37.00 (4.82) | 37.36 (5.27) | 37.19 (5.71) | 36.38 (6.56) |
| Father Education | 3.95 (1.74) | 3.66 (1.63) | 3.37 (1.50) | 3.12 (1.32) |
| Father Health edu | 0.03 (0.18) | 0.03 (0.17) | 0.02 (0.14) | 0.02 (0.13) |
| Father Working | 0.87 (0.34) | 0.85 (0.35) | 0.83 (0.38) | 0.78 (0.41) |
| Father hospital Visits | 0.27 (1.02) | 0.27 (0.89) | 0.30 (1.14) | 0.31 (1.07) |
| Flat | 0.27 (0.44) | 0.23 (0.42) | 0.22 (0.42) | 0.26 (0.44) |
| Home Owner | 0.81 (0.39) | 0.79 (0.41) | 0.76 (0.43) | 0.66 (0.47) |
| Hospital Visits 4-6 | 0.50 (1.84) | 0.40 (1.34) | 0.34 (1.34) | 0.33 (1.20) |
| Household Assets | 21099.86 (52519.53) | 22634.89 (43330.47) | 22324.47 (76167.50) | 19737.34 (53770.00) |
| Household Debts | 33005.15 (29565.89) | 31429.15 (33573.75) | 33178.27 (51069.97) | 37581.08 (66461.62) |
| Household Earnings | 32462.63 (108981.27) | 29574.03 (112921.68) | 25025.51 (104593.33) | 18728.27 (58171.74) |
| Household Insurance income | 521.19 (1651.00) | 514.98 (1651.85) | 573.83 (1748.26) | 825.72 (2003.31) |
| Household Size | 4.52 (1.34) | 4.54 (1.38) | 4.61 (1.53) | 4.53 (1.75) |
| Married Mother | 0.86 (0.35) | 0.84 (0.36) | 0.82 (0.39) | 0.75 (0.43) |
| Missing Father | 0.02 (0.13) | 0.02 (0.13) | 0.02 (0.15) | 0.03 (0.18) |
| Mother Age | 34.85 (4.20) | 35.01 (4.75) | 34.83 (5.32) | 33.94 (6.22) |
| Mother Education | 3.88 (1.57) | 3.68 (1.51) | 3.48 (1.42) | 3.27 (1.30) |
| Mother Health edu | 0.20 (0.40) | 0.18 (0.39) | 0.16 (0.36) | 0.13 (0.33) |
| Mother Hospital Visits | 0.96 (1.26) | 0.95 (1.12) | 0.99 (1.20) | 1.10 (1.28) |
| Mother Working | 0.74 (0.44) | 0.73 (0.44) | 0.70 (0.46) | 0.67 (0.47) |
| Postcode Foreign Born | 0.02 (0.02) | 0.01 (0.01) | 0.01 (0.01) | 0.01 (0.01) |
| Postcode Low Education | 0.41 (0.09) | 0.44 (0.09) | 0.47 (0.09) | 0.48 (0.08) |
| Postcode unemployment | 0.14 (0.09) | 0.14 (0.08) | 0.14 (0.08) | 0.13 (0.07) |
| Remote Area | 2.54 (1.68) | 3.11 (1.96) | 3.61 (2.09) | 3.94 (2.13) |
| Siblings Count | 1.45 (1.01) | 1.48 (1.07) | 1.54 (1.15) | 1.51 (1.24) |
| Single Parent | 0.08 (0.28) | 0.09 (0.28) | 0.11 (0.31) | 0.19 (0.40) |
| Swedish Speaking | 0.05 (0.21) | 0.05 (0.22) | 0.05 (0.21) | 0.04 (0.20) |
| Swedish Speaking Postcode | 0.05 (0.14) | 0.06 (0.16) | 0.05 (0.16) | 0.05 (0.16) |
| Women | 0.43 (0.49) | 0.42 (0.49) | 0.43 (0.49) | 0.42 (0.49) |

Supplementary table 5. The means (standard deviations) of covariates at each ranked CATE fourth for No education. Observations assigned to CATE groups using Causal Forest.

| Variable | 1st CATE Rank | 2nd | 3rd | 4th CATE Rank |
| --- | --- | --- | --- | --- |
| Any Diabetes in Household | 0.04 (0.19) | 0.02 (0.15) | 0.03 (0.17) | 0.02 (0.15) |
| Any Home Care | 0.23 (0.42) | 0.23 (0.42) | 0.23 (0.42) | 0.24 (0.42) |
| Any Pensions income | 0.07 (0.25) | 0.07 (0.26) | 0.10 (0.29) | 0.11 (0.32) |
| Any Social Assistance | 0.13 (0.33) | 0.10 (0.30) | 0.12 (0.32) | 0.12 (0.32) |
| Any Unemployment Benefits | 0.33 (0.47) | 0.33 (0.47) | 0.35 (0.48) | 0.30 (0.46) |
| Birth Year | 1985.63 (2.91) | 1985.80 (2.82) | 1985.72 (2.86) | 1985.84 (2.87) |
| Business Income | 253.80 (1962.12) | 596.03 (2956.77) | 1593.09 (5684.84) | 5163.57 (12644.73) |
| Crowded Household | 0.48 (0.50) | 0.49 (0.50) | 0.51 (0.50) | 0.52 (0.50) |
| Father Age | 37.13 (5.65) | 36.81 (5.39) | 36.68 (5.56) | 37.30 (5.91) |
| Father Education | 3.55 (1.58) | 3.61 (1.59) | 3.46 (1.54) | 3.47 (1.63) |
| Father Health edu | 0.03 (0.16) | 0.03 (0.17) | 0.02 (0.15) | 0.02 (0.14) |
| Father Working | 0.82 (0.38) | 0.85 (0.36) | 0.83 (0.38) | 0.84 (0.37) |
| Father hospital Visits | 0.29 (1.05) | 0.28 (0.99) | 0.29 (1.03) | 0.28 (1.06) |
| Flat | 0.29 (0.45) | 0.22 (0.41) | 0.23 (0.42) | 0.25 (0.43) |
| Home Owner | 0.69 (0.46) | 0.79 (0.41) | 0.77 (0.42) | 0.77 (0.42) |
| Hospital Visits 4-6 | 0.39 (1.49) | 0.40 (1.61) | 0.39 (1.29) | 0.38 (1.39) |
| Household Assets | 19298.77 (33191.31) | 19935.93 (37296.15) | 20206.68 (48152.38) | 26351.20 (92133.08) |
| Household Debts | 23440.48 (40780.32) | 32589.69 (37831.91) | 36406.30 (47330.51) | 42756.78 (59302.72) |
| Household Earnings | 27432.63 (104336.38) | 29134.69 (113451.93) | 25311.03 (121506.41) | 23910.23 (22803.95) |
| Household Insurance income | 743.11 (1971.49) | 597.56 (1760.55) | 559.36 (1695.65) | 535.85 (1643.64) |
| Household Size | 4.47 (1.57) | 4.54 (1.47) | 4.55 (1.48) | 4.64 (1.52) |
| Married Mother | 0.80 (0.40) | 0.84 (0.37) | 0.81 (0.39) | 0.82 (0.38) |
| Missing Father | 0.03 (0.16) | 0.02 (0.14) | 0.02 (0.14) | 0.02 (0.15) |
| Mother Age | 34.83 (5.24) | 34.68 (4.94) | 34.30 (5.08) | 34.81 (5.48) |
| Mother Education | 3.67 (1.41) | 3.75 (1.45) | 3.51 (1.43) | 3.38 (1.55) |
| Mother Health edu | 0.19 (0.39) | 0.20 (0.40) | 0.16 (0.37) | 0.12 (0.33) |
| Mother Hospital Visits | 1.02 (1.21) | 0.98 (1.18) | 1.01 (1.26) | 0.98 (1.22) |
| Mother Working | 0.69 (0.46) | 0.72 (0.45) | 0.70 (0.46) | 0.72 (0.45) |
| Postcode Foreign Born | 0.01 (0.01) | 0.01 (0.01) | 0.01 (0.01) | 0.02 (0.01) |
| Postcode Low Education | 0.44 (0.08) | 0.45 (0.08) | 0.46 (0.09) | 0.46 (0.11) |
| Postcode unemployment | 0.14 (0.08) | 0.14 (0.08) | 0.14 (0.09) | 0.13 (0.08) |
| Remote Area | 2.96 (1.79) | 3.29 (1.94) | 3.43 (2.10) | 3.52 (2.26) |
| Siblings Count | 1.48 (1.15) | 1.48 (1.10) | 1.49 (1.11) | 1.53 (1.12) |
| Single Parent | 0.15 (0.35) | 0.10 (0.30) | 0.12 (0.32) | 0.11 (0.31) |
| Swedish Speaking | 0.04 (0.19) | 0.04 (0.19) | 0.05 (0.21) | 0.06 (0.24) |
| Swedish Speaking Postcode | 0.04 (0.14) | 0.04 (0.15) | 0.05 (0.16) | 0.07 (0.17) |
| Women | 0.42 (0.49) | 0.41 (0.49) | 0.43 (0.49) | 0.43 (0.49) |

Supplementary table 6. The means (standard deviations) of covariates at each ranked CATE fourth for Unemployment Months. Observations assigned to CATE groups using Causal Forest.

| Variable | 1st CATE Rank | 2nd | 3rd | 4th CATE Rank |
| --- | --- | --- | --- | --- |
| Any Diabetes in Household | 0.04 (0.20) | 0.02 (0.13) | 0.02 (0.13) | 0.04 (0.19) |
| Any Home Care | 0.25 (0.43) | 0.23 (0.42) | 0.23 (0.42) | 0.22 (0.41) |
| Any Pensions income | 0.10 (0.30) | 0.08 (0.27) | 0.08 (0.27) | 0.08 (0.28) |
| Any Social Assistance | 0.17 (0.37) | 0.08 (0.28) | 0.09 (0.28) | 0.12 (0.33) |
| Any Unemployment Benefits | 0.37 (0.48) | 0.29 (0.46) | 0.30 (0.46) | 0.35 (0.48) |
| Birth Year | 1986.27 (2.91) | 1985.67 (2.87) | 1985.53 (2.81) | 1985.51 (2.80) |
| Business Income | 2724.95 (10182.52) | 1855.18 (6760.36) | 1659.68 (6268.20) | 1366.72 (5500.25) |
| Crowded Household | 0.49 (0.50) | 0.48 (0.50) | 0.51 (0.50) | 0.51 (0.50) |
| Father Age | 37.58 (6.18) | 37.23 (5.51) | 36.85 (5.32) | 36.27 (5.43) |
| Father Education | 3.46 (1.58) | 3.68 (1.65) | 3.60 (1.62) | 3.35 (1.47) |
| Father Health edu | 0.02 (0.15) | 0.03 (0.17) | 0.03 (0.16) | 0.02 (0.15) |
| Father Working | 0.78 (0.42) | 0.87 (0.33) | 0.86 (0.34) | 0.82 (0.39) |
| Father hospital Visits | 0.32 (1.08) | 0.26 (0.91) | 0.27 (0.97) | 0.30 (1.15) |
| Flat | 0.25 (0.44) | 0.20 (0.40) | 0.23 (0.42) | 0.31 (0.46) |
| Home Owner | 0.75 (0.43) | 0.84 (0.37) | 0.79 (0.40) | 0.64 (0.48) |
| Hospital Visits 4-6 | 0.39 (1.26) | 0.38 (1.35) | 0.41 (1.64) | 0.39 (1.53) |
| Household Assets | 26129.81 (85497.74) | 23471.66 (46886.95) | 21090.57 (52467.30) | 15101.56 (31694.28) |
| Household Debts | 36563.47 (49528.07) | 38877.49 (44215.80) | 34730.16 (51052.19) | 25025.05 (43836.17) |
| Household Earnings | 22073.99 (109165.33) | 29774.63 (108163.78) | 28587.90 (104724.17) | 25356.05 (66617.51) |
| Household Insurance income | 498.82 (1530.07) | 542.94 (1642.23) | 661.57 (1890.05) | 732.57 (1986.16) |
| Household Size | 4.65 (1.62) | 4.67 (1.50) | 4.59 (1.45) | 4.29 (1.43) |
| Married Mother | 0.79 (0.41) | 0.87 (0.34) | 0.85 (0.36) | 0.76 (0.43) |
| Missing Father | 0.03 (0.17) | 0.02 (0.12) | 0.02 (0.13) | 0.03 (0.16) |
| Mother Age | 35.15 (5.56) | 35.08 (5.02) | 34.62 (4.93) | 33.77 (5.12) |
| Mother Education | 3.49 (1.44) | 3.70 (1.51) | 3.64 (1.50) | 3.48 (1.41) |
| Mother Health edu | 0.16 (0.37) | 0.19 (0.39) | 0.17 (0.38) | 0.15 (0.35) |
| Mother Hospital Visits | 1.04 (1.29) | 0.98 (1.16) | 0.99 (1.17) | 0.99 (1.24) |
| Mother Working | 0.63 (0.48) | 0.74 (0.44) | 0.74 (0.44) | 0.71 (0.45) |
| Postcode Foreign Born | 0.02 (0.01) | 0.01 (0.01) | 0.01 (0.01) | 0.01 (0.01) |
| Postcode Low Education | 0.44 (0.09) | 0.45 (0.09) | 0.45 (0.09) | 0.46 (0.10) |
| Postcode unemployment | 0.14 (0.08) | 0.13 (0.08) | 0.13 (0.08) | 0.14 (0.08) |
| Remote Area | 3.04 (1.91) | 3.26 (2.00) | 3.39 (2.08) | 3.53 (2.14) |
| Siblings Count | 1.63 (1.19) | 1.56 (1.11) | 1.50 (1.09) | 1.28 (1.06) |
| Single Parent | 0.16 (0.37) | 0.07 (0.26) | 0.09 (0.28) | 0.15 (0.36) |
| Swedish Speaking | 0.08 (0.26) | 0.05 (0.22) | 0.04 (0.19) | 0.02 (0.14) |
| Swedish Speaking Postcode | 0.08 (0.21) | 0.06 (0.16) | 0.04 (0.13) | 0.03 (0.09) |
| Women | 0.43 (0.49) | 0.42 (0.49) | 0.42 (0.49) | 0.42 (0.49) |

Supplementary table 7. The means (standard deviations) of covariates at each ranked CATE fourth for Income. Observations assigned to CATE groups using Causal Forest.

| Variable | 1st CATE Rank | 2nd | 3rd | 4th CATE Rank |
| --- | --- | --- | --- | --- |
| Any Diabetes in Household | 0.04 (0.20) | 0.02 (0.13) | 0.02 (0.14) | 0.03 (0.18) |
| Any Home Care | 0.25 (0.43) | 0.24 (0.42) | 0.22 (0.42) | 0.21 (0.41) |
| Any Pensions income | 0.09 (0.29) | 0.08 (0.27) | 0.07 (0.26) | 0.07 (0.26) |
| Any Social Assistance | 0.08 (0.27) | 0.12 (0.32) | 0.13 (0.33) | 0.11 (0.31) |
| Any Unemployment Benefits | 0.24 (0.43) | 0.33 (0.47) | 0.36 (0.48) | 0.33 (0.47) |
| Birth Year | 1984.37 (2.57) | 1984.93 (2.52) | 1985.53 (2.45) | 1985.87 (2.42) |
| Business Income | 3062.96 (8889.07) | 1735.41 (6416.54) | 1434.95 (8137.38) | 1136.31 (5302.20) |
| Crowded Household | 0.45 (0.50) | 0.49 (0.50) | 0.52 (0.50) | 0.51 (0.50) |
| Father Age | 36.80 (5.62) | 36.50 (5.46) | 36.77 (5.51) | 37.60 (5.86) |
| Father Education | 3.54 (1.60) | 3.38 (1.48) | 3.41 (1.49) | 3.71 (1.73) |
| Father Health edu | 0.02 (0.16) | 0.02 (0.15) | 0.02 (0.15) | 0.03 (0.16) |
| Father Working | 0.88 (0.33) | 0.83 (0.37) | 0.81 (0.39) | 0.83 (0.38) |
| Father hospital Visits | 0.22 (0.91) | 0.27 (1.04) | 0.30 (1.01) | 0.28 (0.95) |
| Flat | 0.20 (0.40) | 0.24 (0.43) | 0.26 (0.44) | 0.28 (0.45) |
| Home Owner | 0.82 (0.39) | 0.76 (0.43) | 0.73 (0.44) | 0.73 (0.44) |
| Hospital Visits 4-6 | 0.36 (1.37) | 0.40 (1.62) | 0.40 (1.42) | 0.39 (1.44) |
| Household Assets | 30788.26 (78663.78) | 18539.07 (41889.80) | 16466.26 (33411.06) | 16371.35 (35343.87) |
| Household Debts | 39978.06 (48788.26) | 31615.33 (41952.87) | 29387.72 (35684.58) | 33986.26 (46070.34) |
| Household Earnings | 24353.78 (62445.56) | 25289.26 (163289.71) | 24452.75 (16087.35) | 30786.51 (117748.64) |
| Household Insurance income | 845.51 (2090.57) | 622.45 (1725.16) | 506.55 (1585.12) | 495.73 (1682.39) |
| Household Size | 4.72 (1.59) | 4.54 (1.50) | 4.47 (1.47) | 4.46 (1.46) |
| Married Mother | 0.87 (0.34) | 0.82 (0.39) | 0.80 (0.40) | 0.81 (0.39) |
| Missing Father | 0.02 (0.13) | 0.02 (0.15) | 0.02 (0.15) | 0.03 (0.16) |
| Mother Age | 34.32 (5.06) | 34.04 (5.06) | 34.42 (5.13) | 35.57 (5.38) |
| Mother Education | 3.65 (1.50) | 3.45 (1.37) | 3.44 (1.38) | 3.68 (1.57) |
| Mother Health edu | 0.17 (0.37) | 0.15 (0.36) | 0.16 (0.37) | 0.18 (0.38) |
| Mother Hospital Visits | 1.07 (1.12) | 1.02 (1.21) | 0.98 (1.20) | 0.94 (1.19) |
| Mother Working | 0.75 (0.43) | 0.70 (0.46) | 0.69 (0.46) | 0.72 (0.45) |
| Postcode Foreign Born | 0.01 (0.01) | 0.01 (0.01) | 0.01 (0.01) | 0.02 (0.01) |
| Postcode Low Education | 0.46 (0.10) | 0.46 (0.09) | 0.45 (0.09) | 0.44 (0.09) |
| Postcode unemployment | 0.11 (0.09) | 0.13 (0.09) | 0.14 (0.08) | 0.14 (0.07) |
| Remote Area | 3.56 (2.12) | 3.44 (2.04) | 3.29 (2.01) | 2.98 (1.96) |
| Siblings Count | 1.58 (1.17) | 1.49 (1.12) | 1.46 (1.11) | 1.43 (1.08) |
| Single Parent | 0.08 (0.27) | 0.12 (0.32) | 0.13 (0.34) | 0.12 (0.33) |
| Swedish Speaking | 0.04 (0.20) | 0.03 (0.16) | 0.04 (0.19) | 0.08 (0.26) |
| Swedish Speaking Postcode | 0.04 (0.14) | 0.03 (0.12) | 0.05 (0.15) | 0.09 (0.20) |
| Women | 0.42 (0.49) | 0.43 (0.49) | 0.42 (0.49) | 0.42 (0.49) |

1. Rubin DB. Estimating causal effects of treatments in randomized and nonrandomized studies. *Journal of Educational Psychology*. 1974;66(5):688–701.

2. Breiman L. Random Forests. *Machine Learning*. 2001;45(1):5–32.

3. Athey S, Tibshirani J, Wager S. Generalized Random Forests. *The Annals of Statistics*. 2019;47(2):1148–1178.

4. Robinson PM. Root-N-Consistent Semiparametric Regression. *Econometrica*. 1988;56(4):931–954.

5. Robins JM, Rotnitzky A, Zhao LP. Estimation of Regression Coefficients When Some Regressors are not Always Observed. *Journal of the American Statistical Association*. 1994;89(427):846–866.

6. Laing SP, Jones ME, Swerdlow AJ, et al. Psychosocial and Socioeconomic Risk Factors for Premature Death in Young People With Type 1 Diabetes. *Diabetes Care*. 2005;28(7):1618–1623.

7. Secrest AM, Costacou T, Gutelius B, et al. Association of Socioeconomic Status with Mortality in Type 1 Diabetes: The Pittsburgh Epidemiology of Diabetes Complications Study. *Annals of Epidemiology*. 2011;21(5):367–373.

8. Berhan YT, Eliasson M, Möllsten A, et al. Impact of Parental Socioeconomic Status on Excess Mortality in a Population-Based Cohort of Subjects With Childhood-Onset Type 1 Diabetes. *Diabetes Care*. 2015;38(5):827–832.

9. Campbell R a. S, Colhoun HM, Kennon B, et al. Socio-economic status and mortality in people with type 1 diabetes in Scotland 2006–2015: a retrospective cohort study. *Diabetic Medicine*. 2020;37(12):2081–2088.

10. Evans-Cheung TC, Bodansky HJ, Parslow RC, et al. Mortality and acute complications in children and young adults diagnosed with Type 1 diabetes in Yorkshire, UK: a cohort study. *Diabetic Medicine*. 2018;35(1):112–120.

11. Helgeson VS, Wright A, Vaughn A, et al. 14-Year Longitudinal Trajectories of Depressive Symptoms Among Youth With and Without Type 1 Diabetes. *Journal of Pediatric Psychology*. 2022;47(10):1135–1144.

12. Liu S, Leone M, Ludvigsson JF, et al. Association and Familial Coaggregation of Childhood-Onset Type 1 Diabetes With Depression, Anxiety, and Stress-Related Disorders: A Population-Based Cohort Study. *Diabetes Care*. 2022;45(9):1987–1993.

13. Manderbacka K, Sund R, Koski S, et al. Diabetes and depression? Secular trends in the use of antidepressants among persons with diabetes in Finland in 1997–2007. *Pharmacoepidemiology and Drug Safety*. 2011;20(4):338–343.

14. Almeida MC, Claudino DA, Grigolon RB, et al. Psychiatric disorders in adolescents with type 1 diabetes: a case-control study. *Braz. J. Psychiatry*. 2018;40:284–289.

15. Plener PL, Molz E, Berger G, et al. Depression, metabolic control, and antidepressant medication in young patients with type 1 diabetes. *Pediatric Diabetes*. 2015;16(1):58–66.

16. McGrady ME, Hood KK. Depressive symptoms in adolescents with type 1 diabetes: Associations with longitudinal outcomes. *Diabetes Research and Clinical Practice*. 2010;88(3):e35–e37.

17. Helgeson VS, Palladino DK, Reynolds KA, et al. Relationships and health among emerging adults with and without Type 1 diabetes. *Health Psychology*. 2014;33(10):1125–1133.

18. Lind T, Waernbaum I, Berhan Y, et al. Socioeconomic factors, rather than diabetes mellitus per se, contribute to an excessive use of antidepressants among young adults with childhood onset type 1 diabetes mellitus: a register-based study. *Diabetologia*. 2012;55(3):617–624.

19. Sjöberg L, Pitkäniemi J, Haapala L, et al. Fertility in people with childhood-onset type 1 diabetes. *Diabetologia*. 2013;56(1):78–81.

20. Jacobson A m., Hauser S t., Cole C, et al. Social Relationships Among Young Adults with Insulin-dependent Diabetes Mellitus: Ten-year Follow-up of an Onset Cohort. *Diabetic Medicine*. 1997;14(1):73–79.

21. Meo SA, Alkahlan MA, Al-mubarak MA, et al. Impact of type 1 diabetes mellitus on academic performance. *J Int Med Res*. 2013;41(3):855–858.

22. Lindkvist EB, Thorsen SU, Paulsrud C, et al. Association of type 1 diabetes and educational achievement in 16-20-year-olds: A Danish nationwide register study. *Diabet Med*. 2022;39(2):e14673.

23. Thorsted AB, Thygesen LC, Hoffmann SH, et al. Educational outcomes and the role of comorbidity among adolescents with type 1-diabetes in Denmark. *Diabetic Medicine*. n/a(n/a):e15270.

24. Liu S, Ludvigsson JF, Lichtenstein P, et al. Educational Outcomes in Children and Adolescents With Type 1 Diabetes and Psychiatric Disorders. *JAMA Network Open*. 2023;6(4):e238135.

25. Skipper N, Gaulke A, Sildorf SM, et al. Association of Type 1 Diabetes With Standardized Test Scores of Danish Schoolchildren. *JAMA*. 2019;321(5):484–492.

26. Bowden N, Dixon R, Anderson V, et al. Associations between type 1 diabetes and educational outcomes: an Aotearoa/New Zealand nationwide birth cohort study using the Integrated Data Infrastructure. *Diabetologia*. 2024;67(1):62–73.

27. Fleming M, Fitton CA, Steiner MFC, et al. Educational and Health Outcomes of Children Treated for Type 1 Diabetes: Scotland-Wide Record Linkage Study of 766,047 Children. *Diabetes Care*. 2019;42(9):1700–1707.

28. Begum M, Chittleborough C, Pilkington R, et al. Educational outcomes among children with type 1 diabetes: Whole-of-population linked-data study. *Pediatric Diabetes*. 2020;21(7):1353–1361.

29. Cooper MN, McNamara KAR, de Klerk NH, et al. School performance in children with type 1 diabetes: a contemporary population-based study. *Pediatr Diabetes*. 2016;17(2):101–111.

30. Mitchell RJ, McMaugh A, Woodhead H, et al. The impact of type 1 diabetes mellitus in childhood on academic performance: A matched population-based cohort study. *Pediatric Diabetes*. 2022;23(3):411–420.

31. Steen Carlsson K, Landin-Olsson M, Nyström L, et al. Long-term detrimental consequences of the onset of type 1 diabetes on annual earnings—evidence from annual registry data in 1990–2005. *Diabetologia*. 2010;53(6):1084–1092.

32. Matsushima M, Tajima N, Agata T, et al. Social and Economic Impact on Youth-Onset Diabetes in Japan. *Diabetes Care*. 1993;16(5):824–827.
